# New insights into the microbiome of the deep-sea sponge Inflatella pellicula and the secondary metabolic potential of metagenome-assembled genomes and the wider microbiome

**DOI:** 10.1099/mgen.0.001602

**Published:** 2026-01-12

**Authors:** Stephen A. Jackson, Pavlo Hrab, Mitja M. Zdouc, David J. Clarke, Alan D.W. Dobson

**Affiliations:** 1 School of Microbiology, University College Cork, Cork, Ireland; 2 Environmental Research Institute, University College Cork, Cork, Ireland; 3 Laboratory of Microbiology, Wageningen University, Wageningen, Netherlands; 4 Bioinformatics Group, Wageningen University, Wageningen, Netherlands

**Keywords:** deep-sea, metagenome, metagenome-assembled genomes, Porifera, secondary metabolism biosynthetic gene clusters

## Abstract

Marine sponges are found in all of the world’s oceans, from the surface waters to the deepest abyssal zones. The marine sponge holobiont is a rich source of microbial and chemical diversity. Up to 63 bacterial phyla have been observed to be associated with sponges, and thousands of unique natural products have been extracted from sponges or their microbial symbionts. However, sponges from the deep sea and their associated microbial communities are relatively understudied, largely due to sampling-associated difficulties. Secondary metabolism biosynthetic gene clusters are phylogenetically distinct and hold the potential to produce novel chemistry with potential pharmacological or industrial utility. In order to gain further insights into the microbiome of the deep-sea sponge *Inflatella pellicula*, the metagenome of this sponge, sampled from a depth of 2,900 m, was sequenced. A large fraction of the sequence reads appeared to be ‘biological dark matter’ and could not be taxonomically classified. Further, unlike similar studies from different marine ecosystems, relatively few metagenome-assembled genomes (MAGs) could be assembled, and relatively few secondary metabolism biosynthetic gene clusters were identified. The identified clusters were, however, very dissimilar to known characterized clusters, but some shared similarities with clusters annotated in MAGs assembled from sponge metagenomes from disparate geographic locations. Therefore, renewed efforts to cultivate the hosts of these gene clusters may yield valuable small-molecule natural products.

**Impact Statement**

While the deep sea is a relatively underexplored environmental ecosystem, it is known to be inhabited by sponges that host highly diverse symbiotic microbial assemblages. Deep-sea sponge metagenomes are known to contain secondary metabolism biosynthetic gene clusters, which are phylogenetically distant from known and well-characterized metagenomes from both terrestrial and shallow-water environments. This genetic novelty has the capacity to encode microbially derived metabolites with novel chemistry, with the potential for biotechnological exploitation as marine natural products. We report here on the metagenome of *Inflatella pellicula*, sampled from a depth of 2,900 m, a large proportion of which could not be taxonomically classified. Furthermore, secondary metabolism biosynthetic gene clusters in the metagenome displayed very low similarities to clusters encoding known metabolites. These findings highlight the potential of *I. pellicula* and of deep-sea sea sponges in general as potential sources of novel bioactive secondary metabolites.

## Data Summary

The data that support the findings of this study are openly available. The raw sequencing data of the sponge metagenome are available in *National Center for Biotechnology Information* (NCBI) GenBank at https://www.ncbi.nlm.nih.gov/sra/SRX23506756, with the accession number SRX23506756. The secondary metabolism biosynthetic gene cluster dataset used for BiG-SCAPE analysis can be found at https://doi.org/10.5281/zenodo.16792556. The assembled metagenome-assembled genome dataset is available at https://doi.org/10.5281/zenodo.16792619.

## Introduction

Marine sponges (Porifera) are known to play host to diverse communities of microbial symbionts, which proliferate within their mesohyl tissues and, in doing so, make an important functional contribution to the sponge holobiont [1]. These microbial communities, which can be composed of as many as 63 different bacterial phyla, such as *Pseudomonadota* (mainly *Gamma-* and *Alphaproteobacteria*), *Acidobacteriota*, *Chloroflexota*, *Cyanobacteriota* and *Candidatus Poribacteriota*, amongst others, are known to produce a wide range of secondary metabolites, many of which protect the host against predators and epibionts. The enzymes that catalyse the biosynthesis of these bacterial secondary metabolites are typically encoded in biosynthetic gene clusters (BGCs) for pathways involved in the production of polyketides, non-ribosomal peptides, ribosomally synthesized and post-translationally modified peptides (RiPPs) and terpenes [2,4]. These sponge symbionts also play an important role in various metabolic functions within the sponge, including an active role in carbon metabolism particularly involving complex carbohydrates, in nitrogen metabolism, particularly with ammonia oxidation and in vitamin synthesis [5,8].

We have previously investigated the microbial diversity of the deep-sea sponge *Inflatella pellicula* by 16S rRNA gene pyrosequencing (BioSample: SAMN04456058; sample name: BD226 Ip; SRA: SRS1310054) and found it to be host to a unique microbial consortium containing diverse bacteria and archaea, with archaea being present at very high relative abundances within the sponge communities [9,10]. In addition, we subsequently used 454 pyrosequencing targeting both adenylation (AD) and ketosynthase (KS) domain sequences to assess the secondary metabolomic potential of *I. pellicula* (sample name: BD226 Ip; SRA: SRS1310054) [11]. This resulted in AD sequences distantly related to ADs from macrolides (epothilone), lipopeptides (daptomycin) and glycopeptides (vancomycin, bleomycin and balhimycin) biosynthetic gene clusters being identified. In addition, KS sequences from known lipopeptide and macrolide biosynthetic genes were also identified, thereby indicating that polyketide synthase (PKS) and non-ribosomal peptide synthetase (NRPS)-affiliated domains are prevalent amongst the genomes of members of the microbial communities of *I. pellicula* [11].

Cultured bacteria from a number of deep-sea sponges have been reported to display a range of antimicrobial activities against clinically or industrially relevant bacterial pathogens [12,15], while recent reports have also highlighted the high potential for deep-sea sponge-associated bacteria to produce novel natural products [16,18]. Following the analysis of the genomes of 12 bacteria isolated from the deep-sea sponges *Pheronema carpenteri* and *Hertwigia falcifera,* a large number of putative BGCs were identified with low gene similarity to known BGCs [17]; while genome mining of *Streptomyces ortus* isolated from the deep-sea sponge *Polymastia corticata* identified this strain as having a high level of biosynthetic potential [18]. Our group has also previously screened bacteria isolated from the deep-sea sponges, *Lissodendoryx diversichela*, *Stelletta normani* and *I. pellicula* for activity against a range of clinically relevant pathogens and yeasts, and has identified *Streptomyces* sp. B226SN101 from *I. pellicula*, which displayed activity against *Aspergillus fumigatus* [19]. Subsequent analysis of the *Streptomyces* sp. B226SN101 genome revealed the presence of a number of putative secondary metabolism gene clusters, including 2 PKS, 20 NRPS, 4 PKS/NRPS hybrid and 2 bacteriocin clusters. In addition, a number of clusters potentially encoding lantipeptides, siderophores and terpenes were also identified [19]. Thus, it is clear that bacteria from the microbiome of deep-sea sponges are likely to be a good source of bioactive secondary metabolites. Given that the bacteria hosted by these sponges are subjected to quite different environmental conditions such as lower temperatures, higher salinity and pressure together with decreased levels of nutrients and oxygen, than their terrestrial counterparts, or indeed to microbes from shallow water sponges, then it is highly likely that the metabolites which they produce have the potential to be a source of novel chemistry [20,21].

Given that the vast majority of *I. pellicula* symbionts remain uncultured, these symbionts likely constitute a large and as yet untapped reservoir of potentially novel bacterial metabolites. Thus, it can be expected that metagenomic-based approaches would be useful in determining the potential secondary metabolite biosynthetic potential of the *I. pellicula* microbiome, such as the approaches that have recently been employed to explore the chemical diversity of the microbiome of *Mycale hentscheli* [22,23]. In particular, the use of genome-centric approaches employing microbial metagenome-assembled genomes (MAGs) has proven to be useful in uncovering the genetic potential for metabolite production among the uncultured members within various microbial communities [24], such as in studies involving the microbiome of the Great Barrier Reef sponge *Ircinia ramosa* [25] in the deep-sea glass sponge species *Bathydorus* sp. [26], in Antarctic sponges [27] and in sponges from the Atlantic Ocean and the Mediterranean Sea [28].

Thus, we employed a similar approach by sequencing the metagenome of *I. pellicula* to further characterize the natural product biosynthetic potential of the sponge microbiome through the assembly of complete smBGCs from the metagenome and from MAGs derived from the sponge microbiome. We report here that the identified clusters have low similarities to clusters with known bioactive products but share varying levels of similarity to clusters identified in MAGs from diverse sponge metagenomes in disparate geographical locations worldwide. These findings further highlight the potential of deep-sea sponge-associated microbial consortia to produce novel bioactive compounds of potential pharmacological utility.

## Methods

### Sponge sampling

The marine sponge, *I. pellicula* (phylum Porifera, class Demospongiae, order Poecilosclerida, suborder Myxillina, family *Coelospheridae*) was sampled 170 Km off the west coast of Ireland (54.2419 N 12.6938 W), from a depth of 2,900 m, using the Irish research vessel *RV Celtic Explorer* and the ROV *Holland I*. No specific permissions were required as the sample was obtained in Irish waters using an Irish research vessel funded by the Irish government and did not involve endangered or protected sponge species. The sponge sample was immediately rinsed with sterile artificial seawater [3.33 % w/v Instant Ocean (Aquarium Systems – Blacksburg, VA, USA)], placed in a sterile Ziploc bag and frozen at −80 °C until ready for use.

### DNA extraction

Metagenomic DNA was extracted as previously described [9].

### Sequencing

Metagenomic DNA was sequenced commercially, by paired-end (2×150 bp) sequencing using an Illumina NovaSeq 6000 S4 by Eurofins Genomics Ltd, Constance, Germany.

### Sequence processing & analysis

Sequencing data provided by Eurofins was pre-processed by the service provider to remove adapters and for read quality. The read quality of the obtained data was further checked for quality using FastQC v0.11.9 [29]. Reads were assembled using metaSPAdes v3.15.3 [30] with a minimum contig length of 500. Metagenome assemblies were annotated with Prokka v1.14.5 [31] and with RASTtk v1.073 [32]. Sequences were taxonomically classified using Kaiju v1.7.3 [33]. Assembled contigs were binned using CONCOCT v1.1.0 [34] and MetaBAT2 v1.7 [35]. Binned contigs were re-annotated as MAGs using Prokka v1.14.5 and DRAM [36]. MAGs were taxonomically classified using GTDB-Tk v1.7.0 [37]. MAG assembly quality was checked using QUAST v4.4 [38] and CheckM v1.0.18 [39]. All of the above analyses were implemented on the KBase platform [40] using default parameters. Sequencing-effort coverage was estimated using Nonpareil [41] (Fig. S1, available in the online Supplementary Material). Secondary metabolism potential was assessed using antiSMASH v7 beta [42] with relaxed strictness. antiSMASH analyses were performed on the metagenomic assembly and also on the assembled MAGs individually. BGCs detected by antiSMASH version 7.0 in the metagenomic assemblies were clustered based on their sequence similarity using BiG-SCAPE version 1.1.15 [43]. The cut-off value was determined empirically by performing analyses with the cut-off set to 0.3, 0.5 and 0.7. A value of 0.7 was determined to be optimal since it resulted in the maximum number of connections between BGCs. For annotation, BGCs were co-analysed with characterized BGCs from the MIBiG 3.0 database [44]. To determine the distribution of the BGCs in comparison to a larger dataset, cblaster version 1.3.12 [45] was used to match against the National Center for Biotechnology Information (NCBI) NR database (version 20 June 2023), and clinker was used to visualize BGCs aligned to the closest related clusters.

## Results and discussion

### Taxonomic classification of sequencing reads

#### Viruses

Viral sequences comprised 0.273% of taxonomically classified sequencing reads. Of the classified viral reads, 78% recruit to the realms *Riboviria* (49%), *Duplodnaviria* (14.9%) or *Varidnaviria* (14.4%). A further 17.4% of the viral reads were annotated as unclassified phage, while other reads were identified as phages associated with at least 26 bacterial genera. Interestingly, 0.8% of reads classified as viral were identified as pandoraviruses. These viruses are the largest known viruses with genomes of up to 2.5 Mb [46] and are known to infect amoebae. In previous analyses of sponge metatranscriptomes, Pandoraviridae transcripts were found to have two- to threefold higher transcripts per million (TPM) values than the average in *Cymbastela concentrica* and transcribed equal to the average TPM in *Scopalina* sp. Given that pandoraviruses infect amoebae, it has been suggested that they may have the ability to infect amoebocyte cells in sponges [47]. While the roles of bacteria in the sponge holobiont have been extensively studied in recent decades, the influence of viruses and phages in driving and shaping the microbial community structures has to date been largely overlooked [48]. Nonetheless, Pascelli *et al.* used TEM to observe up to 50 different virus-like particle morphologies within sponge cells, in the host mesohyl, on the sponge ectoderm and within sponge-associated microorganisms [49], suggesting an important ecological role for viruses and phages in sponge microbiomes.

#### Archaea

Three per cent of the sequencing reads were taxonomically classified as archaeal, comprising at least 32 archaeal families with ~50% of classified archaeal reads identified as Thaumarchaeota. We have previously reported that the microbiome of the sponge *I. pellicula* was dominated by Archaea [9]. In a pyrosequencing survey, we found that 61% of total reads were archaeal and that 55% of those archaeal reads were from a single operational taxonomic unit (OTU) in the phylum Thaumarchaeota, in the family *Cenarcheaceae*. A possible explanation for the fact that only 3% of the sequencing reads in the present study were classified as *Archaea* is that, with 66% of reads from this study remaining unclassified (Fig. S2), a large proportion may derive from archaea for which, as of yet, no reference sequences are available. The large proportion of sequencing reads that remain unclassified is intriguing. Williams *et al.* did not classify 32.16–56.11 % of reads from deep-sea sponges [50], while Díez-Vives *et al.* were unable to classify 80% of reads from deep-sea sponges [51], providing further proof of the potentially significant value of further analysing deep-sea sponge metagenomes for novel biosynthetic potential. The candidate genus *Nitrosopumilis* (26% of archaeal reads in this study) is frequently and consistently reported to be the dominant archaeal genus in sponges worldwide [52] and is believed to play an important role in host waste processing via ammonia oxidation [53]. Similar to our previous finding, in a recent report, the microbiome of the sponge *Oopsacas minuta* was found to be completely dominated by a single candidate *Cenarchaeum* sp. [54]. The Stenosarchaea group accounted for 32% of classified archaeal reads, with 52.8% of those classified in the halophilic class Halobacteria and 47% in the methanogenic class Methanomicrobia.

#### Bacteria

Of the classified reads, 2.43% were assigned to the domain Bacteria but could not be assigned to any phylum. Taxonomically classified bacterial sequence reads assigned to phyla at relative abundances ≥0.5% represented 13 phyla (Fig. S2). Of those classified reads, 14% could not be assigned to a family, 38% represented families present at relative abundances of ≥0.5%, while the remainder were assigned to 47 bacterial families (Fig. S3). The metabolically talented phylum *Actinobacteriota* accounted for 13% of classified bacterial reads and was represented by 164 genera. The genus *Streptomyces* accounted for 12.5% of actinobacterial reads and 1.64% of the total bacterial reads. Although the phylum *Chloroflexota* accounted for 4.1% of the classified bacterial reads, and the class *Dehalococcoidia* accounted for just 18.9% of those (0.8% of all classified bacterial sequences), four of the eight MAGs reported here were from this class (Table 1). Similarly, despite the phylum *Acidobacteriota* accounting for just 2.3% of classified bacterial reads, a near high-quality MAG was assembled from this phylum. High-quality assemblies are defined as >90 % completion, <5 % contamination [55]. Surprisingly, despite MAGs being obtained from the candidate phylum *Poribacteria* and from *Tectomicrobia* (Table 1), the Kaiju classifier did not assign any sequence reads to those taxa.

**Table 1.** Taxonomic assignment, completeness and contamination of medium- and high-quality MAGs from the metagenome of *I. pellicula*

Bin ID	Taxonomy	MAG completeness (%)	MAG contamination (%)
B226_SM_CONCOCT_bin.001	d__Bacteria;p__Tectomicrobia;c__Entotheonellia;o__Entotheonellales;f__Entotheonellaceae;g__SXND01;s__	63.4	1.71
B226_SM_CONCOCT_bin.018	d__Bacteria;p__Chloroflexota;c__Dehalococcoidia;o__UBA3495;f__UBA3495;g__Casp-Chloro-G3;s__	66.94	3.96
B226_SM_CONCOCT_bin.020	d__Bacteria;p__Poribacteria;c__WGA-4E;o__WGA-4E;f__WGA-3G;g__WGA-3G;s__	91.4	2.35
B226_SM_CONCOCT_bin.023	d__Bacteria;p__Chloroflexota;c__Dehalococcoidia;o__UBA3495;f__UBA3495;g__VXOI01;s__	88.25	0.25
B226_SM_CONCOCT_bin.025	d__Bacteria;p__Proteobacteria;c__Alphaproteobacteria;o__UBA828;f__UBA828;g__WTGU01;s__	77.47	2.09
B226_SM_metaBAT2_bin.006	d__Bacteria;p__Chloroflexota;c__Dehalococcoidia;o__UBA3495;f__UBA3495;g__Bin87;s__	85.63	0
B226_SM_metaBAT2_bin.008	d__Bacteria;p__Chloroflexota;c__Dehalococcoidia;o__UBA3495;f__UBA3495;g__Bin87;s__	84.62	0.33
B226_SM_metaBAT2_bin.011	d__Bacteria;p__Acidobacteriota;c__Acidobacteriae;o__VXMN01;f__VXMN01;g__VXMN01;s__	83.69	0

### Metagenome assembled genomes

Sequencing reads binned using CONCOCT and metaBAT2 were manually dereplicated. Of the 45 bins produced by CONCOCT and 18 bins produced by metaBAT2, after dereplication, only medium-quality (≥50% completion, <10% contamination) and high-quality (>90 % completion, <5% contamination) MAGs [55] were retained for further analysis (Table 1). This resulted in the further analysis of eight MAGs (Table S2). As previously mentioned, four of these MAGs were recruited to the phylum *Chloroflexota*. *Chloroflexota* comprised 4.1% of taxonomically classified reads from the metagenome. One MAG was classified as a member of the phylum *Acidobacteriota*, which was represented by 3.8% of classified reads in the metagenome. One MAG was identified as an *Alphaproteobacterium*, which was the most abundant class amongst taxonomically classified reads in the metagenome. The remaining MAGs were classified as *Poribacteria* and *Tectomicrobia* despite no sequence reads being classified in those phyla by the Kaiju or GOTTCHA2 classifiers.

### Secondary metabolism biosynthetic gene clusters

The potential of the sponge microbiome, and of the assembled MAGs within the microbiome, to produce novel natural products was assessed using BiG-SCAPE, antiSMASH, cblaster and clinker. antiSMASH allows for the identification and annotation of smBGCs and identifies the closest related clusters with known and characterized metabolic products through the Minimum Information about a Biosynthetic Gene Cluster (MIBiG) dataset, while cblaster identifies the closest related clusters annotated in GenBank but which may or may not be further characterized. Here, the closest related clusters identified by cblaster were used to create linkage maps using clinker (Figs S4-S7).

The metagenome and the assembled MAGs were analysed separately using antiSMASH. The metagenome contained 17 smBGCs (Table 2). The low number of clusters observed was a somewhat unexpected result. While antiSMASH only accepts assembled contigs ≥1 Kb in length as input, only 23% of contigs assembled here met that cut-off (Table S2); nonetheless, the total length of assembled contigs here was 47% of the total assembly length, and similarly sized sponge-derived metagenomes have been reported to contain orders of magnitude more smBGCs [23,28, 56]. Additionally, short-read sequencing of metagenomes can lead to imperfect assemblies, which makes confident interpretations of BGC lengths, completeness and characteristics challenging. Following antiSMASH analysis of the individual high- and medium-quality MAGs, 9 of the 17 smBGCs identified in the metagenome could be assigned to those MAGs (Table 2). These clusters appeared to be unique with little similarity to known clusters in the antiSMASH reference dataset. Further, the highest similarity scores to known clusters in the MIBiG database, which links clusters to experimentally validated known metabolites, ranged from 0.05 to 0.35 (where 1.0=100 %). Similarity scores from cblaster analyses, where metabolic products are largely unknown, ranged from 0.05 to 0.51%.

**Table 2.** Secondary metabolism gene clusters assigned to assembled MAGs or unassigned

	Taxonomic source	Cluster type	Length (bp)
1	*CONCOCT_bin.020* d__Bacteria;p__Poribacteria;c__WGA-4E;o__WGA-4E;f__WGA-3G;g__WGA-3G;s__	RRE-containing	13,772
2	*CONCOCT_bin.020* d__Bacteria;p__Poribacteria;c__WGA-4E;o__WGA-4E;f__WGA-3G;g__WGA-3G;s__	NRPS-like	12,254
3	*CONCOCT_bin.023* d__Bacteria;p__Chloroflexota;c__Dehalococcoidia;o__UBA3495;f__UBA3495;g__VXOI01;s__	Terpene	14,694
4	*CONCOCT_bin.023* d__Bacteria;p__Chloroflexota;c__Dehalococcoidia;o__UBA3495;f__UBA3495;g__VXOI01;s__	Phosphonate	7,424
5	*metaBAT2_bin.006* _Bacteria;p__Chloroflexota;c__Dehalococcoidia;o__UBA3495;f__UBA3495;g__Bin87;s__	Terpene	18,928
6	*metaBAT2_bin.006* d__Bacteria;p__Chloroflexota;c__Dehalococcoidia;o__UBA3495;f__UBA3495;g__Bin87;s__	Phosphonate	17,418
7	*metaBAT2_bin.008* d__Bacteria;p__Chloroflexota;c__Dehalococcoidia;o__UBA3495;f__UBA3495;g__Bin87;s__	Phosphonate	16,168
8	*metaBAT2_bin.008* d__Bacteria;p__Chloroflexota;c__Dehalococcoidia;o__UBA3495;f__UBA3495;g__Bin87;s__	Terpene	23,482
9	*metaBAT2_bin.011* d__Bacteria;p__Acidobacteriota;c__Acidobacteriae;o__VXMN01;f__VXMN01;g__VXMN01;s__	NRPS-like	13,993
10	Unassigned	T3PKS	33,777
11	Unassigned	Betalactone	18,560
12	Unassigned	RRE-containing	20,313
13	Unassigned	Terpene	18,498
14	Unassigned	Terpene	12,221
15	Unassigned	RiPP-like	7,335
16	Unassigned	T3PKS	10,678
17	Unassigned	T3PKS	10,469

RRE, RiPP recognition element.

### PKS clusters

Polyketide synthase gene clusters were observed in the sponge metagenome but not in any of the MAGs assembled from the metagenome. While only type III PKS clusters were identified, the lack of annotated type I PKS clusters was somewhat surprising, particularly as type I ketosynthase genes were identified in this sponge by Borchert *et al.* [11] and also type I PKS clusters were identified in the genome of *Streptomyces* sp. B226SN101, which was isolated from this sponge [19]. It must be assumed that the lack of type I PKS clusters here is the result either of sequence assembly deficiencies or of sequencing coverage limitations.

**Fig. 1.**
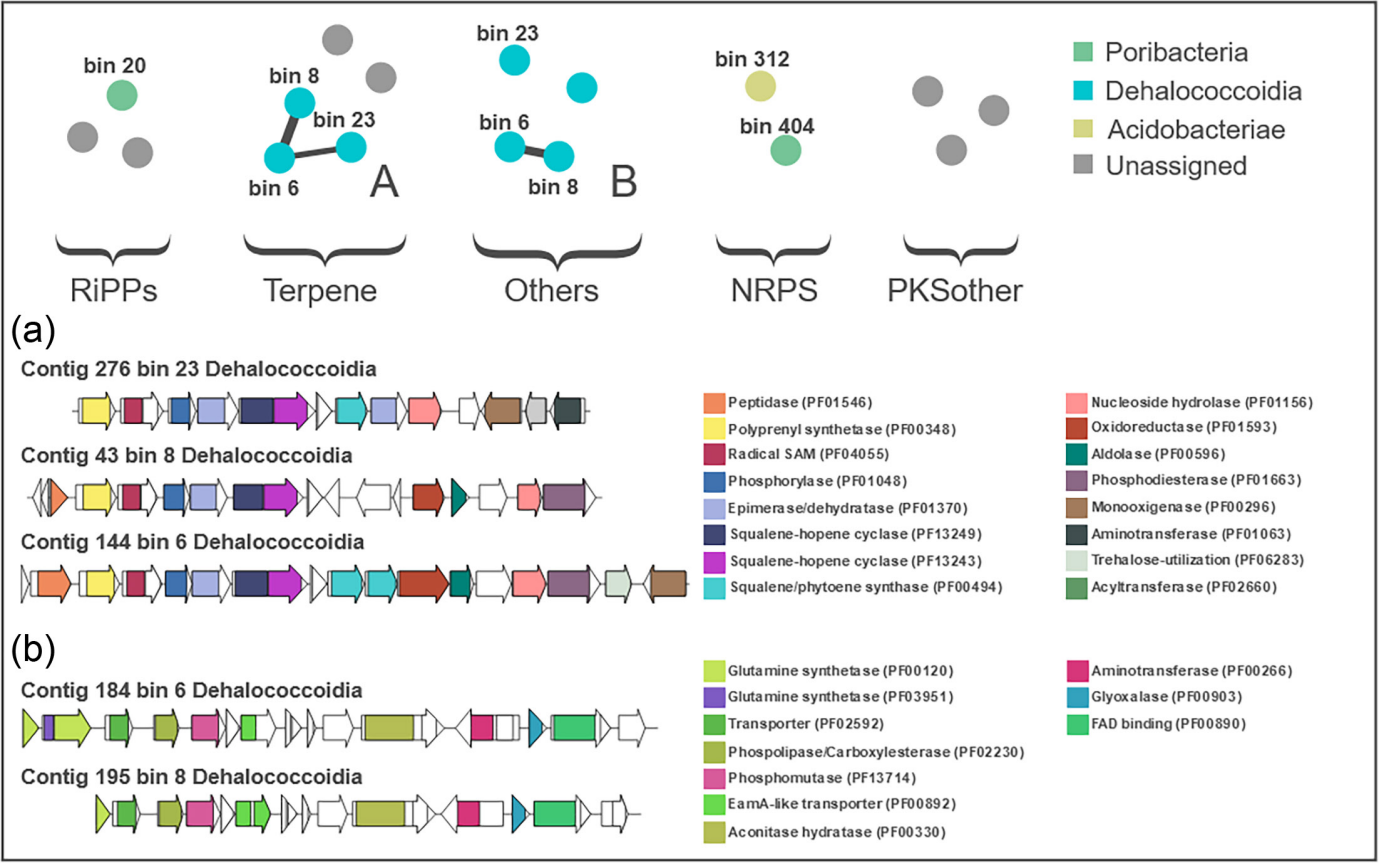
BiG-SCAPE analysis of smBGCs from the metagenome of *I. pellicula*. The upper panel displays network analysis and taxonomic assignment of cluster families. The lower panel shows alignments of cluster families where network connections were observed and Pfam identities. (a) Alignment and annotation of terpene clusters where network connections were observed. (b) Alignment and annotation of phosphonate clusters where network connections were observed.

The three type III PKS clusters observed in the *I. pellicula* metagenome displayed low similarities (similarity scores 0.05, 0.21 and 0.26) to clusters linked to known secondary metabolites. Similarity network analysis revealed that they were unlike each other (Fig. 1).

When compared to BGCs with experimentally validated metabolic products in the MIBiG database, the PKS clusters identified here were most similar to clusters from *Streptomyces* sp. smBGC (MIBiG Cluster BGC0000286), *Streptacidiphilus oryzae* (BGC0002656) and *Cyanobium* sp. (BGC0001962), which produce viguiepinol [57]; oryzanaphthopyrans, oryzanthrones and chlororyzanthrones [58]; and hierridins [59], respectively. However, cblaster analysis of the clusters reveals the closest related clusters to be a type III PKS cluster from the MAG of a *Dadabacterium* sp. genome assembled from the metagenome of the coral reef sponge *Ircinia ramosa* (Fig. S4A, Table S1, node_5), a cluster from an archaeal MAG (*Cenarchaeum* sp.), assembled from the sponge *Theonella swinhoei* (Fig. S4B, Table S1, node_536) and an *Acidobacteriota* MAG assembled from the tropical sponge *Coscinoderma mathewsi* (Fig. S4C, Table S1, node_557), respectively.

Multiple *Dadabacteria* MAGS were previously reconstructed from the Tara Oceans global marine metagenomic samples [55], and subsequent reports indicate that they are likely to be heterotrophic oligotrophs that have the capacity to degrade microbially derived dissolved organic matter such as phospholipids and peptidoglycan [60]. They have also recently been reported in bacterial communities from calcareous sponges sampled off the island of Rodrigues, in the Indian Ocean [61]. Interestingly, type III PKS clusters are reported to be common in ‘organic carbon-associated’ *Dadabacterium* MAGs but were not identified in pelagic *Dadabacterium* MAGs, suggesting a possible symbiosis-related function [60]. PKS clusters are exceptionally rare in archaeal genomes [62], and it is still unclear whether or not archaea can biosynthesize polyketides [63].

Considering the low similarities between the PKS clusters identified here and those with known metabolic products, it can be presumed that they have the potential to produce novel secondary metabolites. Notably, the closest related clusters all derive from MAGs assembled from sponge metagenomes, highlighting the importance of these as reservoirs of genetic diversity for the discovery of potential novel bioactive molecules.

### RiPPs and NRPS

Five smBGCs identified in the metagenome were classified as RiPPs (including RiPP recognition element (RRE) containing) or NRPS. In similarity network analysis, neither the RiPPs clusters nor the NRPS clusters displayed connections to each other (Fig. 1). Furthermore, only low similarities to known clusters in the MIBiG database (similarity scores 0.12–0.27) were observed.

When compared to BGCs in MIBiG, the peptide producing clusters were most similar to clusters which produce the nucleoside antibiotics angustmycins [64] and aristeromycin [65] from *Streptomyces angustmyceticus* (BGC0002407 and BGC0002621), the toyoncin producing cluster from *Bacillus toyonensis* [66] (BGC0002442), the alkylpyrone producing cluster (BGC0001831) from *Myxococcus xanthus* [67], the chuangxinmycin producing biosynthetic gene cluster from *Actinoplanes tsinanensis* (BGC0001485) [15] and the fatty acid-derived ether lipids (VEPE, AEPE and TG-1) producing cluster from *Myxococcus xanthus* (BGC0000871).

Two of the above BGCs were assigned to an individual high-quality *Poribacteria* MAG in the candidate genus WGA-3G [clusters 1 and 2, Table 2 (Table S1, node_131 and node_404)] assembled here, but the closest known clusters identified by cblaster are from two distinct *Poribacteria* MAGs derived from different sponge species [*Pseudoceratina* sp. (Fig. S5B) and *Ircinia ramosa* (Fig. S5E)] sampled from locations thousands of kilometres apart (Guam and Great Barrier Reef). *Poribacteria* are widespread in sponges, and while they have not, as yet, been cultivated, they are known to comprise a significant proportion of the biomass of the holobiont [68]. Single-cell genomics [69] and MAG assemblies [70] have been employed to gain insights into the putative lifestyle of the phylum and have revealed that unique smBGCs are common in *Poribacteria*. These include unusually small, phylogenetically distinct SUP-type PKS clusters [69], and, in addition, phylogenetic analysis of poribacterial fatty acid synthases has shown that they form a distinct phylogenetic clade [2]. This may indicate that the phylum has the potential to produce novel secondary metabolites of interest and also that this biodiscovery potential is widely distributed. The intensification of efforts to cultivate *Poribacteria* may have high potential to yield pharmaceutically important metabolites.

Another NRPS cluster identified here was assigned in this study to an *Acidobacteriota* MAG, in the genus VXMN01 [cluster 9, Table 2 (Table S1, node_312)]. *Acidobacteriota* are both commonly found and abundant in sponge microbiomes, with the phylum being identified as potential ‘super producers’ of natural products based on the BGC content of their genomes (including MAGs) [28]. cblaster analysis revealed that the closest related cluster is an NRPS cluster in an *Acidobacteriota* MAG assembled from the sponge *Ircinia ramosa* (Fig. S5C).

The remaining two peptide-producing BGCs identified in the *I. pellicula* metagenome [clusters 12 and 15, Table 2 (Table S1, node_100 and node_557)] were not assigned to MAGs assembled here. The closest related clusters identified by cblaster were from a ‘Thermoproteota archaeon’ (Fig. S5A) MAG assembly from the biofilm on a filter membrane in a water desalination and re-use plant and from a *Nitrospira* sp. MAG (Fig. S5D) assembled from the metagenome of the marine sponge *Coscinoderma mathewsi*.

### Terpenes

While pharmaceutically relevant terpenoids are typically produced by plants or fungi, smBGCs encoding terpenes are, however, widespread in bacterial genomes (Rudolf et al., 2021). In bacteria, terpenes are synthesized by the mevalonate pathway, where different terpene precursors, isopentenyl diphosphate and dimethylallyl diphosphate, are catalysed into isoprene units of different lengths by prenyltransferases and then cyclized by terpene synthases to produce a range of structurally diverse terpenes [71]. Of all the smBGCs identified in this study, the clusters encoding terpenes displayed the highest similarities to known clusters (similarity scores 0.27–0.35) with known metabolites from the MIBiG database and also the highest inter-cluster family similarities in network analysis (Fig. 1). The terpene clusters showing network connections to each other (clusters 3, 5 and 8, Table 2; bins 6, 8 and 23 Fig. 1) could be assigned to MAGs derived from two genera (bin 87 and VXOI01) of *Dehalococcoidia* in the phylum *Chloroflexota* (Table 2). The other two terpenes could not be assigned to a taxonomic source using read binning programmes.

MIBiG searches identified a carotenoid-producing cluster from *Myxococcus xanthus* (MIBiG Cluster BGC0000648) to be most similar to both clusters 3 and 8 (Table 2) (Table S1, node_43 and node_276). In this study, these terpene BGCs were assigned to two individual MAGs in different candidate genera (Bin_87 and VXOI01) in the phylum *Chloroflexota*. A phytoene-producing cluster from *Rhodobacter sphaeroides* (BGC0000647) (Table S1, node_144; Fig. S6B) was the most similar to cluster 5 (Table 2). The remaining two terpene clusters (clusters 13 and 14, Table 2) identified in the *I. pellicula* metagenome were most similar to the sodorifen-producing cluster from *Serratia* sp. and the carotenoid cluster from *R. sphaeroides* (BGC0002283 and BGC0000647, respectively) (Table S1, node_157 and node_341).

When considering the most similar gene clusters from cblaster searches, were clusters from MAGs assembled from the metagenomes of the marine sponge *Ircinia ramosa*, a ‘marine metagenome’ from seawater sampled from a depth of 50 m, and from the metagenome of sediment from the Mariana Trench (Table S1, Fig. S6A-E).

### Other smBGCs

Four other clusters encoding potential smBGCs, including one betalactone encoding cluster and three phosphonate clusters, were also identified. Betalactone natural products are chemically very diverse with over 30 core scaffolds being reported to date, with some having antibacterial and antifungal activities, while others have displayed strong bioactivity against human cancer cell lines [72]. Despite their valuable therapeutic functions, and while the NRPS-like modular assembly of betalactones has been described, as a class of natural products, they remain largely underexplored, and their biosynthesis is not fully understood [73].

The betalactone encoding cluster (cluster 11, Table 2) from the *I. pellicula* metagenome was most similar, in the MIBiG database, to the dipeptide antibiotic bacilysin, producing cluster (BGC0000888) from *Bacillus* sp. CS93 [74]. The *Inflatella*-derived betalactone cluster showed high similarities to clusters from *Dadabacteria* (Fig. S7A; Table S1, node_8). ORF6 (ctg8_6) in the *I. pellicula*-derived cluster is an 87 aa hypothetical protein, and similarity searches using blastp only returned eight results, indicating the uniqueness of this gene and the possibility that it encodes a presumably rare function. The search results, from alpha- and betaproteobacteria, an acidobacterium and a firmicute, are also hypothetical proteins showing 36–46 % sequence identity.

Bioactive phosphonate natural products are, like betalactones, relatively understudied. They can inhibit important enzyme activities by competitive binding to proteins with structural analogues as native substrates [75]. Antibacterial, antifungal, antiparasitic and herbicidal compounds have been reported. Three phosphonate smBGCs were identified from the *I. pellicula* metagenome. All three phosphonate smBGCs identified here were assigned to three different *Chloroflexota* MAGs assembled in this study (clusters 4, 6 and 7, Table 2). The two largest phosphonate smBGCs (Fig. S7B and C, clusters 6 and 7, Table 2) share similarities, and network connections were identified by BiG-SCAPE (Fig. 1, bins 6 and 8), but differences are also apparent. The closest related clusters in MIBiG analysis identify the same BGC as being the most closely related cluster (BGC0001683). This BGC, which displays low similarities (similarity score: 0.16) to both *I. pellicula* phosphonate clusters, is an *N*-acyl serinol-producing gene cluster from *Bacillus* sp. 2_A_57_CT2 [76]. cblaster searches revealed that the most similar cluster is from a *Chloroflexota* MAG assembled from the metagenome of the sponge *Aplysina aerophoba* (Table S1, node_184 and node_195). The third phosphonate smBGC (cluster 4, Table 2) is unlike the previous two. This 7.4 Kb cluster comprises just five genes, including the characteristic core phosphoenolpyruvate phosphomutase. The closest related cluster with a known metabolic product from MIBiG is the FR-900098 antibiotic-producing cluster from *Streptomyces rubellomurinus* (BGC0000904) [77]. Using cblaster, the closest related cluster is from a *Chloroflexota* bacterium MAG assembled from the metagenome of *Ircinia ramosa* (Fig. S7D, Table S1, node_803).

### Symbiosis-related factors in the *I. pellicula* metagenome

Symbiosis between sponges and microbes is a well-established concept [78] with the microbes performing vital metabolic functions for the host and the sponge providing a niche habitat for the microbe. Microbial assemblages in marine sponges are stable and sponge species-specific [79]. The metazoan host must discriminate between commensal microbes and those that are a food source; therefore, recognition factors must be presented by the microbes [80]. Additionally, accessory genes and related functions must be present in the microbiome to fulfil the commensal functions. Here, we identified the presence of genes related to carbon, nitrogen and sulphur metabolism in the sponge metagenome and in the assembled MAGs, as evidence for potential cross-feeding and waste processing activities. Clustered Regularly Interspaced Short Palindromic Repeats (CRISPR) and toxin/antitoxin genes involved in defence and persistence in MAGs assembled from the metagenome and from the entire metagenome were also identified.

### Carbon metabolism

Evidence for the presence of a variety of carbon metabolism pathways was identified in the sponge metagenome and in the assembled MAGs, with the three main central carbon metabolism pathways (Embden–Meyerhof pathway, the pentose phosphate pathway (PPP) and tricarboxylic acid cycle) being present in the sponge metagenome (Fig. S8A). These pathways were also present in all the MAGs except for the Entotheonella_SXND01 MAG (Table 1), which lacked the Entner–Doudoroff pathway. That MAG assembly was only 63.4% complete; nonetheless, Lackner and colleagues made a similar observation in their analysis of genomes (MAGs) from the order *Entotheonellales* [81]. Similarly, the pathway for 2-carbon metabolism (glyoxylate pathway) was absent from the *Entotheonella* assembly but present in the metagenome and in all other MAGs (Fig. S8A). Evidence for carbon fixation was present in the sponge metagenome and in the assembled MAGs. Genes involved in the reductive-PPP cycle, the dicarboxylate–hydroxybutyrate pathway, the hydroxypropionate–hydroxybutyrate cycle, the 3-hydroxypropionate bi-cycle, the Wood–Ljungdahl pathway and methanogenesis were present in the MAGs from Proteobacteria_WTGUO1 and Acidobacteria_VXMN01 (Fig. S8A). The *Chloroflexota* MAGs (Bin87_MAG_6, Bin87_Mag_7 and Casp-Chloro_G3) hosted all of these C-fixation pathways except for the 3-hydroxypropionate bi-cycle. The latter was also absent from the MAGs of Entotheonella_SXND01 and Poribacteria WGA-3G. The acetyl-CoA carbon assimilation (CO_2_ → acetyl-CoA) pathway was, however, absent from the metagenome and from all MAGs.

### Nitrogen metabolism

Inconsistencies were observed in the identification of the presence of nitrogen metabolism-related genes in the sponge metagenome and in the MAGs assembled from the metagenome. While genes involved in assimilatory and dissimilatory nitrate reduction were observed in MAGs (Fig. S8B), some genes were not observed in the wider metagenome. No complete nitrate reduction pathway was observed in any individual MAG. Similarly, genes for denitrification were observed in five of the eight MAGs, but no MAG hosted a complete pathway. Genes for nitrogen fixation were identified in the MAGs of Entotheonella_SXND01 and *Poribacteria* WGA-3G. Surprisingly, some genes from these pathways, while observed in MAGs, were not annotated in the metagenome. Incomplete N-metabolism pathways in single genomes are not rare [82] and indicate that effective nitrogen catabolism and fixation processes may be the result of a community effort.

### Sulphur metabolism

Similar to nitrogen metabolism pathways, sulphur metabolism genes were identified in MAGs (Fig. S4C) that were not identified in the metagenome. Additionally, as before, no complete pathways were observed in an individual MAG. Specifically, no assimilatory sulphate reduction genes for the conversion of sulphate to adenosine 5′-phosphosulphate (APS) were observed in all MAGs except for those of Chloroflexota _Casp-Chloro_G3 and Acidobacteria_VXMN01. Genes for the conversion of APS to 3′-phosphoadenylyl sulphate (PAPS) were absent from the metagenome and from all MAGs. However, genes for the conversion of PAPS to sulphite (*cysH*) were observed in the genome of Entotheonella_SXND01. Sulphite reduction genes [sulphite reductase (*sir*)] were present in the metagenome and in all MAGs except for those of Entotheonella_SXND01 and Proteobacteria_WTGU01. Notwithstanding that no method to convert APS to PAPS was identified for assimilatory sulphate reduction, genes from the dissimilatory sulphate reduction pathway (*aprAB*) can reduce APS to sulphite, thus allowing complete reduction to proceed. In the dissimilatory pathway, sulphate adenylyltransferase (*sat*) was identified in six of the eight MAGs (Fig. S8C), and the aforementioned *aprAB* genes are present in four of the eight MAGs, allowing for the reduction of sulphate to sulphide. Dissimilatory sulphide reduction genes (*dsr*) were identified in the metagenome but were absent from all MAGs. As previously mentioned, complete reduction can be achieved by *sir* genes (sulphite reductase) from the assimilatory pathway. All genes for thiosulphate oxidation were observed in the metagenome (*soxABCDGHSXYZ*) (Fig. S8C).

### Defence-related factors

Toxin–antitoxin (TA) systems in bacteria play a vital role in defence through various mechanisms, including suicide cell-death mediators under stressful conditions, bacteriostatic effectors of dormancy, so-called plasmid addiction and anti-addiction modules and in host defence against phage attack [83]. CRISPR-Cas systems are primarily an adaptive immune mechanism in bacteria that protect against invading foreign mobile genetic elements and other DNA, such as phages [84]. Together, TA and CRISPR systems play important roles in helping to shape microbial community structures.

Components of 12 different cognate TA systems were observed in the sponge metagenome and in each of the 8 MAGs assembled here (Fig. S9). However, only seven instances of complete cognate pairs were observed in the MAGs, while 15 instances of only one of either the toxin or the antitoxin were observed. CRISPR genes were present in four of the eight MAGs.

### Summary

The deep sea is a largely underexplored habitat, hosting microbial and genetic dark matter that holds the potential for the biodiscovery of novel biosynthetic pathways that produce natural products only distantly related to known entities. Small molecules produced by taxa from the deep sea may find invaluable utility in pharmacy or industry. Marine sponges are an ideal biological enrichment system for microbial genetic material. Here, we reveal that the metagenome of the deep-sea sponge *I. pellicula* is unique and harbours fewer identifiable secondary metabolism biosynthetic gene clusters than might be expected based on similar studies of other sponge species. Furthermore, the clusters that were identified are only distantly related to characterized gene clusters, although some show high degrees of similarity to other uncharacterized clusters present in the metagenomes of other deep-sea sponges with diverse global distributions.

## Supplementary material

10.1099/mgen.0.001602 Uncited Supplementary Material 1.

## Abbreviations

AD: adenylation
APS: adenosine 5′-phosphosulphate
BGCs: biosynthetic gene clusters
KS: ketosynthase
MAGs: metagenome-assembled genomes
MIBiG: Minimum Information about a Biosynthetic Gene Cluster
PAPS: 3′-phosphoadenylyl sulphate
PPP: pentose phosphate pathway
RiPPs: ribosomally synthesized and post-translationally modified peptides
TA: toxin–antitoxin
TPM: transcripts per million

## References

[1] Webster NS, Thomas T (2016). The sponge hologenome. *mBio*.

[2] Dat TTH, Steinert G, Cuc NTK, Cuong PV, Smidt H (2022). Diversity of bacterial secondary metabolite biosynthetic gene clusters in three vietnamese sponges. Mar Drugs.

[3] Medema MH, Kottmann R, Yilmaz P, Cummings M, Biggins JB (2015). Minimum information about a biosynthetic gene cluster. Nat Chem Biol.

[4] Wang S, Li X, Yang W, Huang R (2024). Exploring the secrets of marine microorganisms: unveiling secondary metabolites through metagenomics. Microb Biotechnol.

[5] Fan L, Reynolds D, Liu M, Stark M, Kjelleberg S (2012). Functional equivalence and evolutionary convergence in complex communities of microbial sponge symbionts. Proc Natl Acad Sci USA.

[6] Moitinho-Silva L, Díez-Vives C, Batani G, Esteves AI, Jahn MT (2017). Integrated metabolism in sponge-microbe symbiosis revealed by genome-centered metatranscriptomics. ISME J.

[7] Pita L, Rix L, Slaby BM, Franke A, Hentschel U (2018). The sponge holobiont in a changing ocean: from microbes to ecosystems. Microbiome.

[8] Slaby BM, Hackl T, Horn H, Bayer K, Hentschel U (2017). Metagenomic binning of a marine sponge microbiome reveals unity in defense but metabolic specialization. ISME J.

[9] Jackson SA, Flemer B, McCann A, Kennedy J, Morrissey JP (2013). Archaea appear to dominate the microbiome of *Inflatella pellicula* deep sea sponges. PLoS One.

[10] Kennedy J, Flemer B, Jackson SA, Morrissey JP, O’Gara F (2014). Evidence of a putative deep sea specific microbiome in marine sponges. PLoS One.

[11] Borchert E, Jackson SA, O’Gara F, Dobson ADW (2016). Diversity of natural product biosynthetic genes in the microbiome of the deep sea sponges *Inflatella pellicula*, *Poecillastra compressa*, and *Stelletta normani*. Front Microbiol.

[12] Back CR, Stennett HL, Williams SE, Wang L, Ojeda Gomez J (2021). A new *Micromonospora* strain with antibiotic activity isolated from the microbiome of a mid-atlantic deep-sea sponge. Mar Drugs.

[13] Oluwabusola ET, Jackson SA, Brunati C, Gackstatter S, Vedder H (2025). Integrated omics-based discovery of bioactive halogenated metabolites from the deep-sea *Streptomyces* sp. B188M101. *Mar Drugs*.

[14] Williams SE, Stennett HL, Back CR, Tiwari K, Ojeda Gomez J (2020). The bristol sponge microbiome collection: a unique repository of deep-sea microorganisms and associated natural products. Antibiotics (Basel).

[15] Xu X, Zhou H, Liu Y, Liu X, Fu J (2018). Heterologous Expression Guides Identification of the Biosynthetic Gene Cluster of Chuangxinmycin, an Indole Alkaloid Antibiotic. J Nat Prod.

[16] Chen L, Liu K, Hong J, Cui Z, He W (2024). The discovery of weddellamycin, a tricyclic polyene macrolactam antibiotic from an antarctic deep-sea-derived *Streptomyces* sp. DSS69, by heterologous expression. *Mar Drugs*.

[17] Hesketh-Best PJ, January GG, Koch MJ, Warburton PJ, Howell KL (2023). Whole genomes of deep-sea sponge-associated bacteria exhibit high novel natural product potential. FEMS Microbes.

[18] Williams SE, Back CR, Best E, Mantell J, Stach JEM (2023). Discovery and biosynthetic assessment of “*Streptomyces ortus*” sp. nov. isolated from a deep-sea sponge. Microb Genom.

[19] Jackson SA, Crossman L, Almeida EL, Margassery LM, Kennedy J (2018). Diverse and abundant secondary metabolism biosynthetic gene clusters in the genomes of marine sponge derived *Streptomyces* spp. isolates. *Marine Drugs*.

[20] Afoullouss S, Sanchez AR, Jennings LK, Kee Y, Allcock AL (2022). Unveiling the chemical diversity of the deep-sea sponge *Characella pachastrelloides*. Mar Drugs.

[21] Steffen K, Indraningrat AAG, Erngren I, Haglöf J, Becking LE (2022). Oceanographic setting influences the prokaryotic community and metabolome in deep-sea sponges. Sci Rep.

[22] Rust M, Helfrich EJN, Freeman MF, Nanudorn P, Field CM (2020). A multiproducer microbiome generates chemical diversity in the marine sponge *Mycale hentscheli*. Proc Natl Acad Sci USA.

[23] Storey MA, Andreassend SK, Bracegirdle J, Brown A, Keyzers RA (2020). Metagenomic exploration of the marine sponge *Mycale hentscheli* uncovers multiple polyketide-producing bacterial symbionts. mBio.

[24] Setubal JC (2021). Metagenome-assembled genomes: concepts, analogies, and challenges. Biophys Rev.

[25] Engelberts JP, Robbins SJ, de Goeij JM, Aranda M, Bell SC (2020). Characterization of a sponge microbiome using an integrative genome-centric approach. ISME J.

[26] Wei TS, Gao ZM, Gong L, Li QM, Zhou YL (2023). Genome-centric view of the microbiome in a new deep-sea glass sponge species *Bathydorus* sp. Front Microbiol.

[27] Moreno-Pino M, Manrique-de-la-Cuba MF, López-Rodríguez M, Parada-Pozo G, Rodríguez-Marconi S (2024). Unveiling microbial guilds and symbiotic relationships in Antarctic sponge microbiomes. Sci Rep.

[28] Loureiro C, Galani A, Gavriilidou A, Chaib de Mares M, van der Oost J (2022). Comparative metagenomic analysis of biosynthetic diversity across sponge microbiomes highlights metabolic novelty, conservation, and diversification. mSystems.

[29] Andrews S (2010). FastQC: A Quality Control Tool for High Throughput Sequence Data [Online]. http://www.bioinformatics.babraham.ac.uk/projects/fastqc/.

[30] Nurk S, Meleshko D, Korobeynikov A, Pevzner PA (2017). metaSPAdes: a new versatile metagenomic assembler. Genome Res.

[31] Seemann T (2014). Prokka: rapid prokaryotic genome annotation. Bioinformatics.

[32] Brettin T, Davis JJ, Disz T, Edwards RA, Gerdes S (2015). RASTtk: A modular and extensible implementation of the RAST algorithm for building custom annotation pipelines and annotating batches of genomes. Sci Rep.

[33] Menzel P, Ng KL, Krogh A (2016). Fast and sensitive taxonomic classification for metagenomics with Kaiju. Nat Commun.

[34] Alneberg J, Bjarnason BS, de Bruijn I, Schirmer M, Quick J (2014). Binning metagenomic contigs by coverage and composition. Nat Methods.

[35] Kang DD, Froula J, Egan R, Wang Z (2015). MetaBAT, an efficient tool for accurately reconstructing single genomes from complex microbial communities. PeerJ.

[36] Shaffer M, Borton MA, McGivern BB, Zayed AA, La Rosa SL (2020). DRAM for distilling microbial metabolism to automate the curation of microbiome function. Nucleic Acids Res.

[37] Chaumeil PA, Mussig AJ, Hugenholtz P, Parks DH (2019). GTDB-Tk: a toolkit to classify genomes with the genome taxonomy database. Bioinformatics.

[38] Gurevich A, Saveliev V, Vyahhi N, Tesler G (2013). QUAST: quality assessment tool for genome assemblies. Bioinformatics.

[39] Parks DH, Imelfort M, Skennerton CT, Hugenholtz P, Tyson GW (2015). CheckM: assessing the quality of microbial genomes recovered from isolates, single cells, and metagenomes. Genome Res.

[40] Arkin AP, Cottingham RW, Henry CS, Harris NL, Stevens RL (2018). KBase: The United States department of energy systems biology knowledgebase. Nat Biotechnol.

[41] Rodriguez-R LM, Konstantinidis KT (2014). Nonpareil: a redundancy-based approach to assess the level of coverage in metagenomic datasets. Bioinformatics.

[42] Blin K, Shaw S, Augustijn HE, Reitz ZL, Biermann F (2023). antiSMASH 7.0: new and improved predictions for detection, regulation, chemical structures and visualisation. Nucleic Acids Res.

[43] Navarro-Muñoz JC, Selem-Mojica N, Mullowney MW, Kautsar SA, Tryon JH (2020). A computational framework to explore large-scale biosynthetic diversity. Nat Chem Biol.

[44] Terlouw BR, Blin K, Navarro-Muñoz JC, Avalon NE, Chevrette MG (2023). MIBiG 3.0: a community-driven effort to annotate experimentally validated biosynthetic gene clusters. Nucleic Acids Res.

[45] Gilchrist CLM, Booth TJ, van Wersch B, van Grieken L, Medema MH (2021). cblaster: a remote search tool for rapid identification and visualization of homologous gene clusters. *Bioinform Adv*.

[46] Yong E (2013). Giant viruses open Pandora’s box. Nature.

[47] Nguyen M, Wemheuer B, Laffy PW, Webster NS, Thomas T (2021). Taxonomic, functional and expression analysis of viral communities associated with marine sponges. PeerJ.

[48] Breitbart M, Bonnain C, Malki K, Sawaya NA (2018). Phage puppet masters of the marine microbial realm. Nat Microbiol.

[49] Pascelli C, Laffy PW, Kupresanin M, Ravasi T, Webster NS (2018). Morphological characterization of virus-like particles in coral reef sponges. PeerJ.

[50] Williams SE, Varliero G, Lurgi M, Stach JEM, Race PR (2024). Diversity and structure of the deep-sea sponge microbiome in the equatorial Atlantic Ocean. Microbiology.

[51] Díez-Vives C, Riesgo A (2024). High compositional and functional similarity in the microbiome of deep-sea sponges. ISME J.

[52] Turon M, Uriz MJ (2020). New insights into the archaeal consortium of tropical sponges. Front Mar Sci.

[53] Hoffmann F, Radax R, Woebken D, Holtappels M, Lavik G (2009). Complex nitrogen cycling in the sponge *Geodia barretti*. Environ Microbiol.

[54] Santini S, Schenkelaars Q, Jourda C, Duchesne M, Belahbib H (2023). The compact genome of the sponge *Oopsacas minuta* (Hexactinellida) is lacking key metazoan core genes. BMC Biol.

[55] Bowers RM, Kyrpides NC, Stepanauskas R, Harmon-Smith M, Doud D (2017). Minimum information about a single amplified genome (MISAG) and a metagenome-assembled genome (MIMAG) of bacteria and archaea. Nat Biotechnol.

[56] El Samak M, Zakeer S, Hanora A, Solyman SM (2023). Metagenomic and metatranscriptomic exploration of the Egyptian red sea sponge *Theonella* sp. associated microbial community. Mar Genomics.

[57] Hernández-Falcón J, Taboada J, Guerrero C, Campos-Lozada V, Fernández D (1991). Relaxant effect of viguiepinol on smooth muscle in vitro. Proc West Pharmacol Soc.

[58] Chen S, Zhang C, Zhang L (2022). Investigation of the molecular landscape of bacterial aromatic polyketides by global analysis of type II polyketide synthases. Angew Chem Int Ed.

[59] Costa M, Sampaio-Dias IE, Castelo-Branco R, Scharfenstein H, Rezende de Castro R (2019). Structure of hierridin C, Synthesis of hierridins B and C, and evidence for prevalent alkylresorcinol biosynthesis in picocyanobacteria. J Nat Prod.

[60] Graham ED, Tully BJ (2021). Marine dadabacteria exhibit genome streamlining and phototrophy-driven niche partitioning. ISME J.

[61] Cleary DFR, Oliveira V, Gomes NCM, Bialecki A, de Voogd NJ (2023). A comparison of free-living and sponge-associated bacterial communities from a remote oceanic island with a focus on calcareous sponges. FEMS Microbiol Ecol.

[62] Wang H, Fewer DP, Holm L, Rouhiainen L, Sivonen K (2014). Atlas of nonribosomal peptide and polyketide biosynthetic pathways reveals common occurrence of nonmodular enzymes. Proc Natl Acad Sci USA.

[63] McBride CM, Miller EL, Charkoudian LK (2025). An updated catalogue of diverse type II polyketide synthase biosynthetic gene clusters captured from large-scale nucleotide databases. Microb Genom.

[64] Shiraishi T, Xia J, Kato T, Kuzuyama T (2021). Biosynthesis of the nucleoside antibiotic angustmycins: identification and characterization of the biosynthetic gene cluster reveal unprecedented dehydratase required for exo-glycal formation. J Antibiot.

[65] Yu L, Zhou W, She Y, Ma H, Cai YS (2021). Efficient biosynthesis of nucleoside cytokinin angustmycin A containing an unusual sugar system. Nat Commun.

[66] Wang J, Xu H, Liu S, Song B, Liu H (2021). Toyoncin, a novel leaderless bacteriocin that is produced by *Bacillus toyonensis* XIN-YC13 and specifically targets B. cereus and *Listeria monocytogenes*. Appl Environ Microbiol.

[67] Hug JJ, Panter F, Krug D, Müller R (2019). Genome mining reveals uncommon alkylpyrones as type III PKS products from myxobacteria. J Ind Microbiol Biotechnol.

[68] Fieseler L, Horn M, Wagner M, Hentschel U (2004). Discovery of the novel candidate phylum “Poribacteria” in marine sponges. Appl Environ Microbiol.

[69] Siegl A, Kamke J, Hochmuth T, Piel J, Richter M (2011). Single-cell genomics reveals the lifestyle of Poribacteria, a candidate phylum symbiotically associated with marine sponges. ISME J.

[70] González-Castillo A, Carballo JL, Bautista-Guerrero E (2021). Genomics and phylogeny of the proposed phylum “Candidatus Poribacteria” associated with the excavating sponge Thoosa mismalolli. Antonie Van Leeuwenhoek.

[71] Cheng S, Wang X, Deng Z, Liu T (2025). Innovative approaches in the discovery of terpenoid natural products. Curr Opin Microbiol.

[72] Robinson SL, Christenson JK, Wackett LP (2019). Biosynthesis and chemical diversity of β-lactone natural products. Nat Prod Rep.

[73] Džunková M, La Clair JJ, Tyml T, Doud D, Schulz F (2023). Synthase-selected sorting approach identifies a beta-lactone synthase in a nudibranch symbiotic bacterium. Microbiome.

[74] Moran S, Robertson K, Paradisi F, Rai DK, Murphy CD (2010). Production of lipopeptides in *Bacillus* sp. CS93 isolated from Pozol. FEMS Microbiol Lett.

[75] Polidore ALA, Furiassi L, Hergenrother PJ, Metcalf WW (2021). A phosphonate natural product made by pantoea ananatis is necessary and sufficient for the hallmark lesions of onion center rot. mBio.

[76] Cohen LJ, Esterhazy D, Kim S-H, Lemetre C, Aguilar RR (2017). Commensal bacteria make GPCR ligands that mimic human signalling molecules. Nature.

[77] Eliot AC, Griffin BM, Thomas PM, Johannes TW, Kelleher NL (2008). Cloning, expression, and biochemical characterization of *Streptomyces rubellomurinus* genes required for biosynthesis of antimalarial compound FR900098. Chem Biol.

[78] Taylor MW, Radax R, Steger D, Wagner M (2007). Sponge-associated microorganisms: evolution, ecology, and biotechnological potential. Microbiol Mol Biol Rev.

[79] Lee OO, Wang Y, Yang J, Lafi FF, Al-Suwailem A (2011). Pyrosequencing reveals highly diverse and species-specific microbial communities in sponges from the Red Sea. ISME J.

[80] Wilkinson CR, Garrone R, Vacelet J (1984). Marine sponges discriminate between food bacteria and bacterial symbionts: electron microscope radioautography and *in situ* evidence. Proc R Soc Lond B.

[81] Lackner G, Peters EE, Helfrich EJN, Piel J (2017). Insights into the lifestyle of uncultured bacterial natural product factories associated with marine sponges. Proc Natl Acad Sci USA.

[82] Albright MBN, Timalsina B, Martiny JBH, Dunbar J (2019). Comparative genomics of nitrogen cycling pathways in bacteria and archaea. Microb Ecol.

[83] Jurėnas D, Fraikin N, Goormaghtigh F, Van Melderen L (2022). Biology and evolution of bacterial toxin-antitoxin systems. Nat Rev Microbiol.

[84] Zaayman M, Wheatley RM (2022). Fitness costs of CRISPR-Cas systems in bacteria. Microbiology.

