# Supplementary material for: New insights into the microbiome of the deep-sea sponge Inflatella pellicula and the secondary metabolic potential of metagenome-assembled genomes and the wider microbiome
Source: Microb Genom. 2026 Jan 12;12(1):001602. doi: 10.1099/mgen.0.001602 (PMC12795557; doi:10.1099/mgen.0.001602)
Supplement: Uncited Supplementary Material 1. [file mgen-12-01602-s001.pdf]

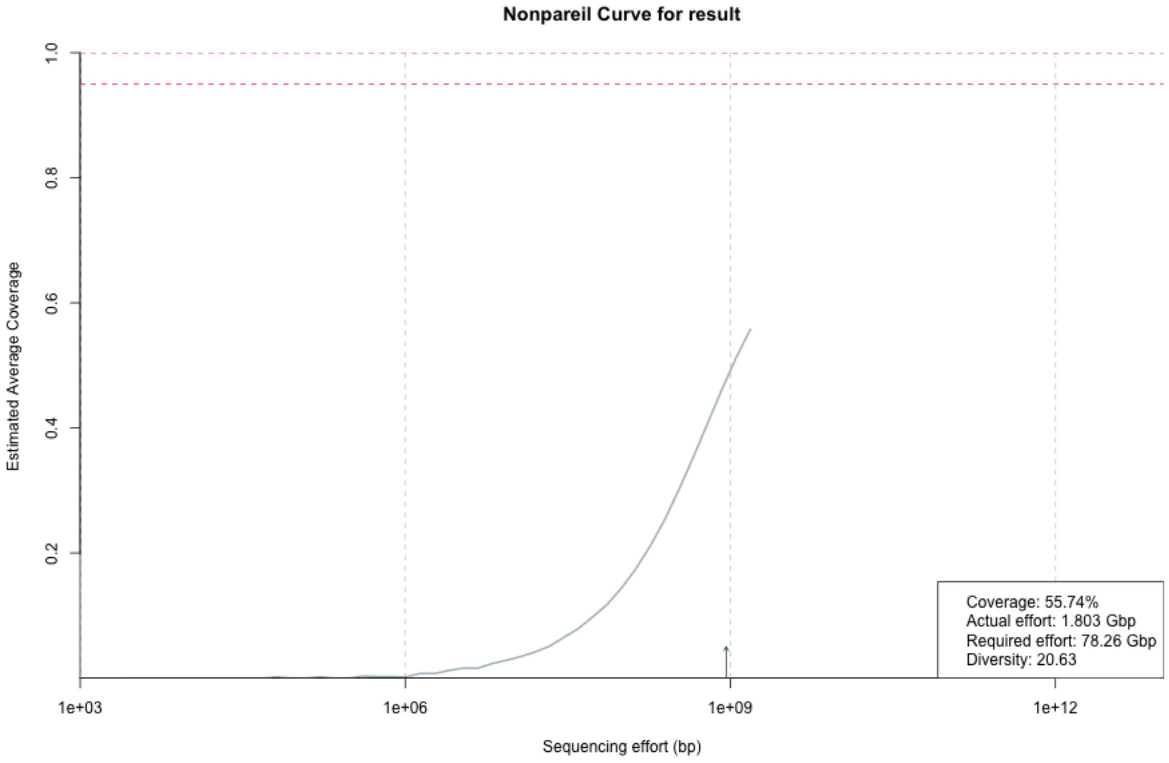

Supplementary Figure S1: Estimated metagenome sequencing and diversity coverage by the sequencing effort achieved.

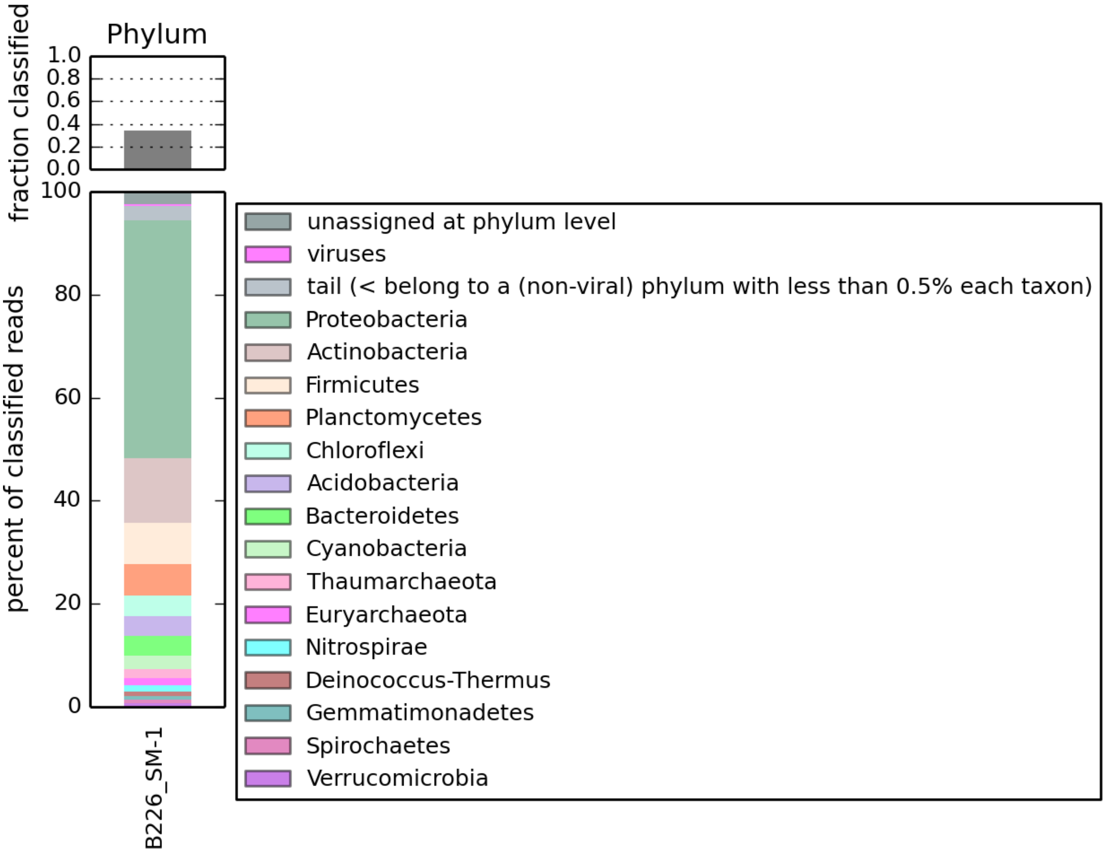

Supplementary Figure S2: Phylum level taxonomic classification of metagenomic sequencing reads from *Inflatella pellicula.*

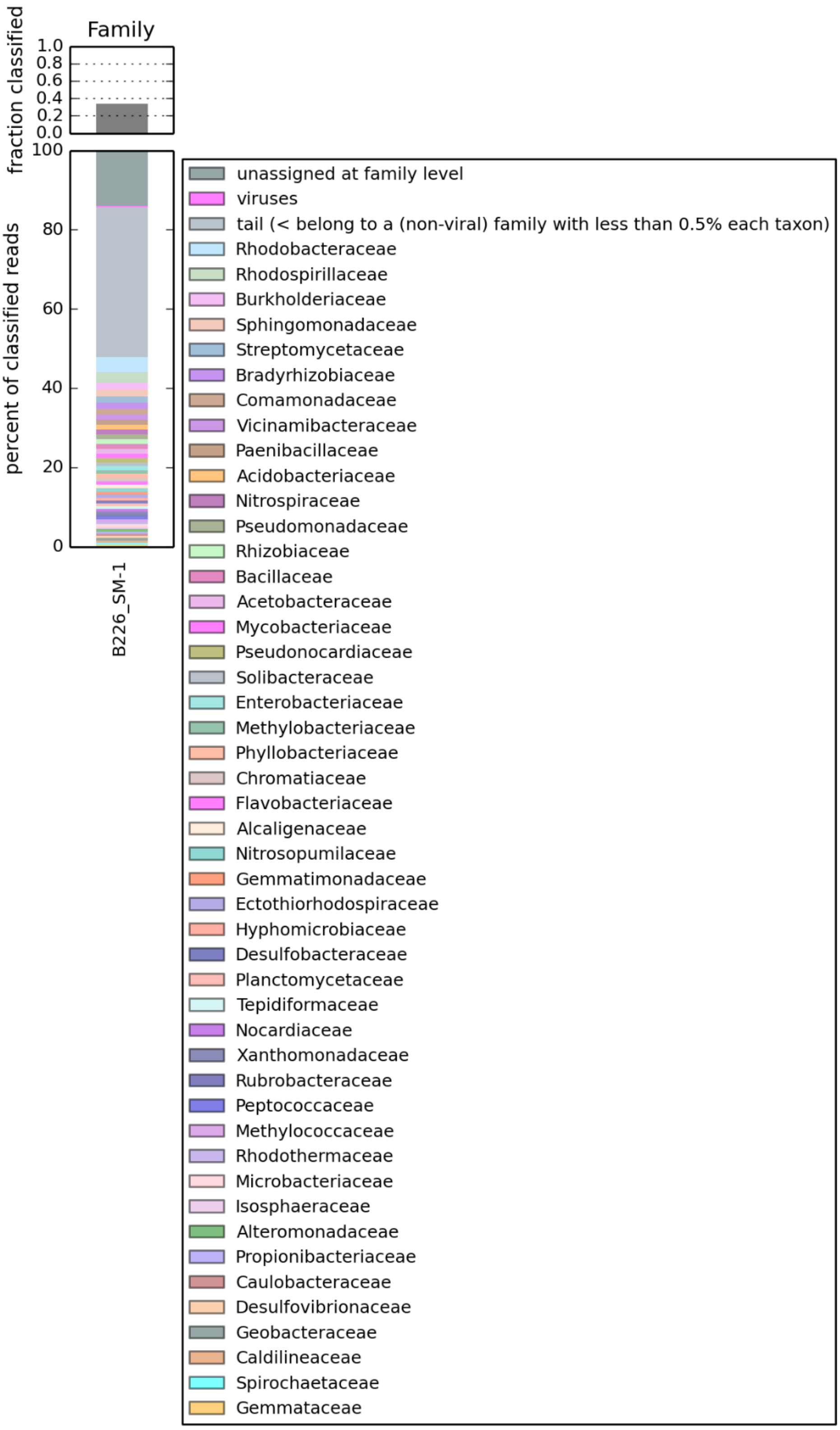

Supplementary Figure S3: Family level taxonomic classification of metagenomic sequencing reads from *Inflatella pellicula.*

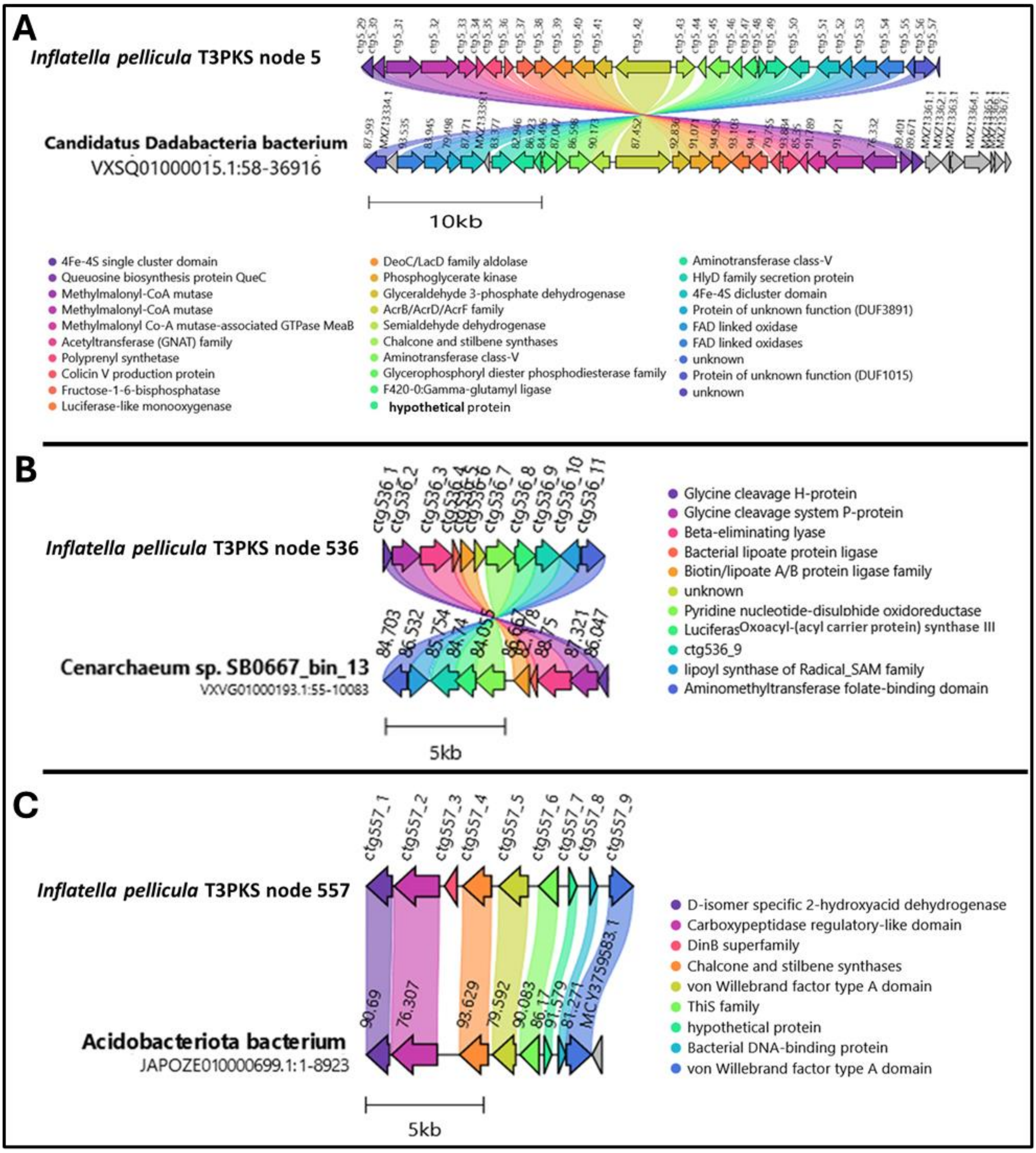

Supplementary Figure S4: Alignment and annotation of PKS clusters from the metagenome of *I. pellicula* and the closest related clusters as identified by cblaster analysis. Where homologous genes were present, percentage identities are indicated, where no homologous genes were present GenBank accession numbers are indicated.

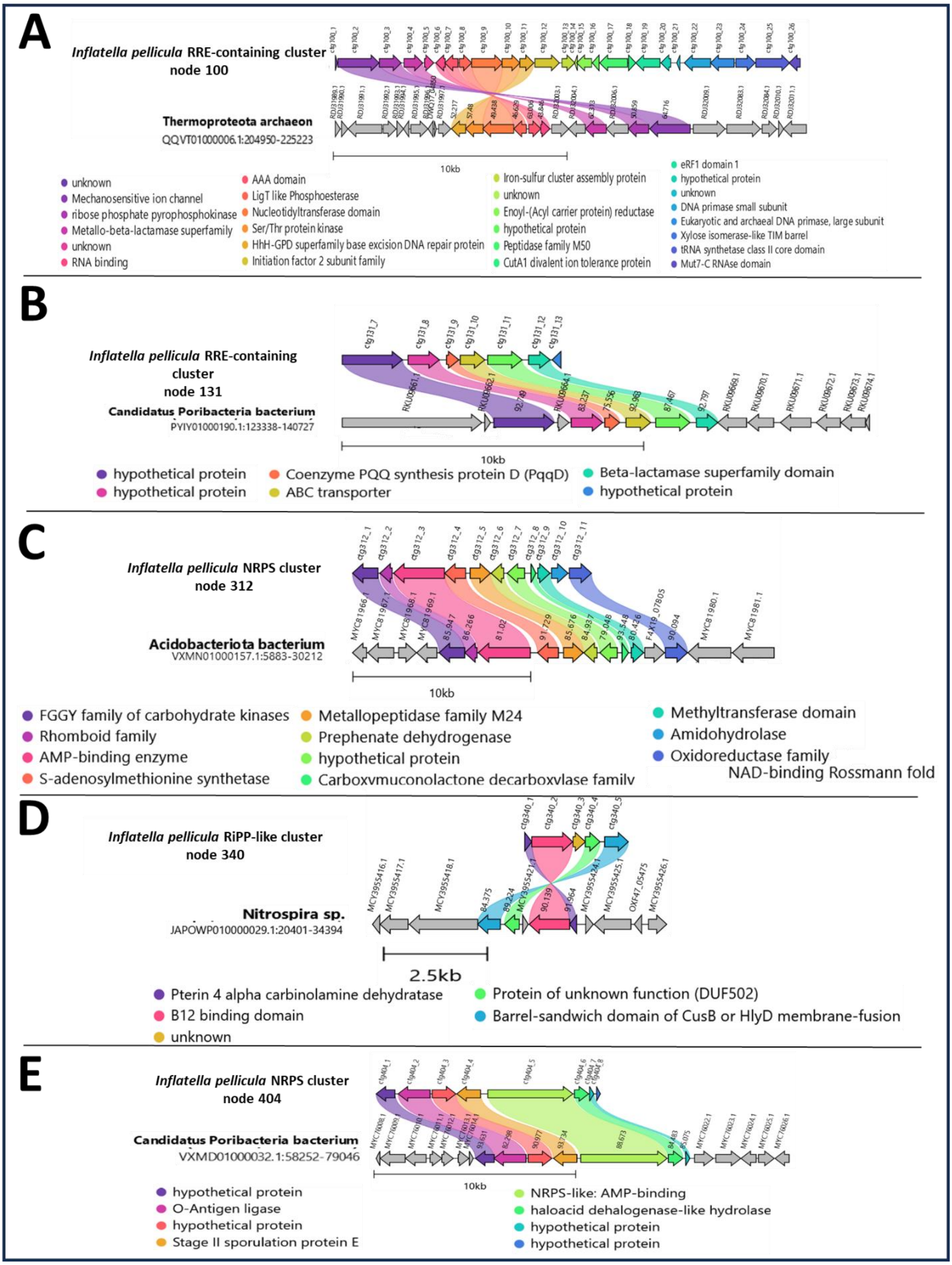

Supplementary Figure S5: Alignment and annotation of RRE-containing (A and B), NRPS (C and E) or RiPP-like (D) secondary metabolism gene clusters from the metagenome of *I. pellicula* and the closest related clusters as identified by cblaster analysis. Where homologous genes were present, percentage identities are indicated, where no homologous genes were present GenBank accession numbers are indicated.

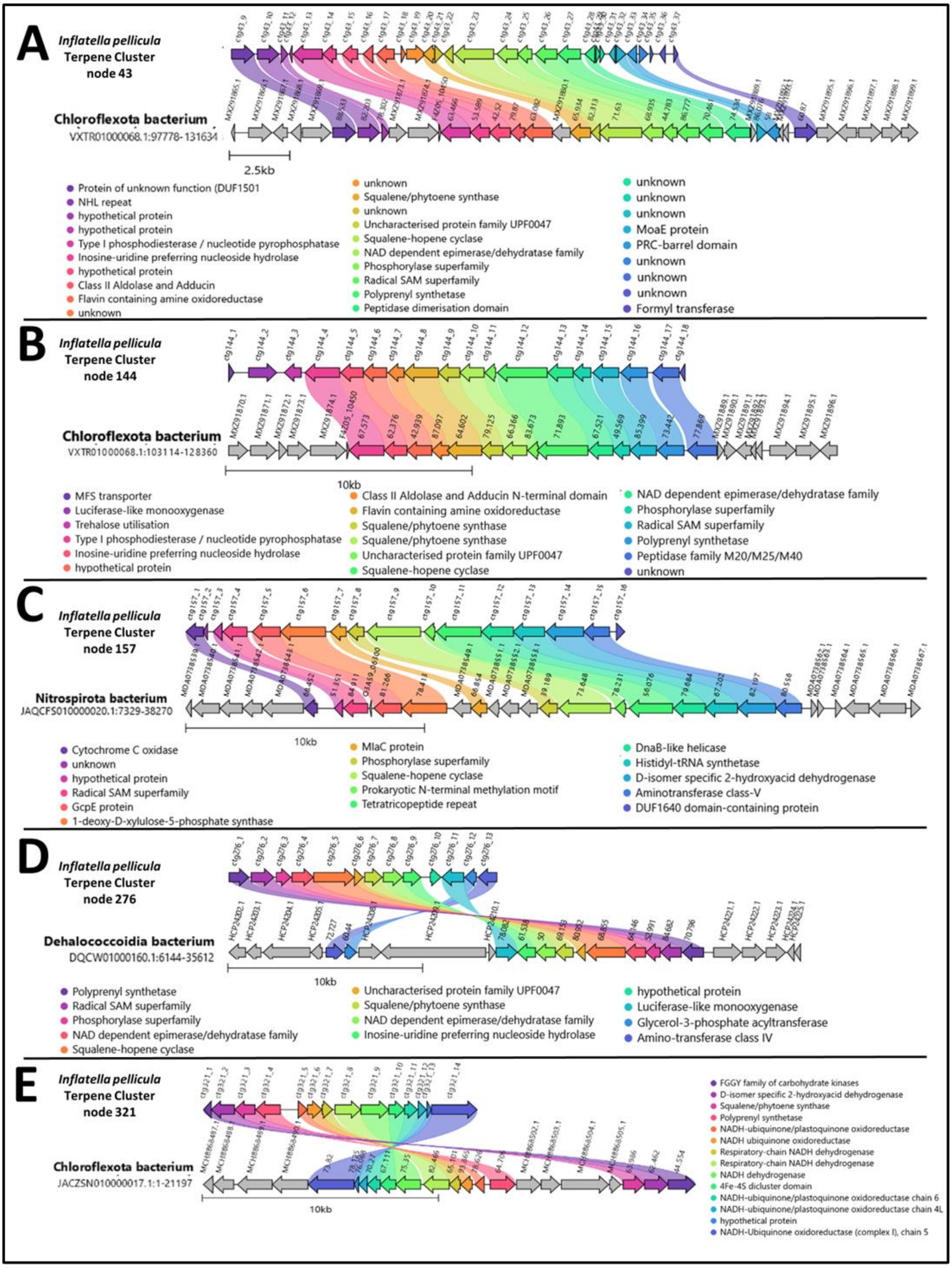

Supplementary Figure S6: Alignment and annotation of terpene-encoding secondary metabolism gene clusters from the metagenome of *I. pellicula* and the closest related clusters as identified by cblaster analysis. Where homologous genes were present, percentage identities are indicated, where no homologous genes were present GenBank accession numbers are indicated.

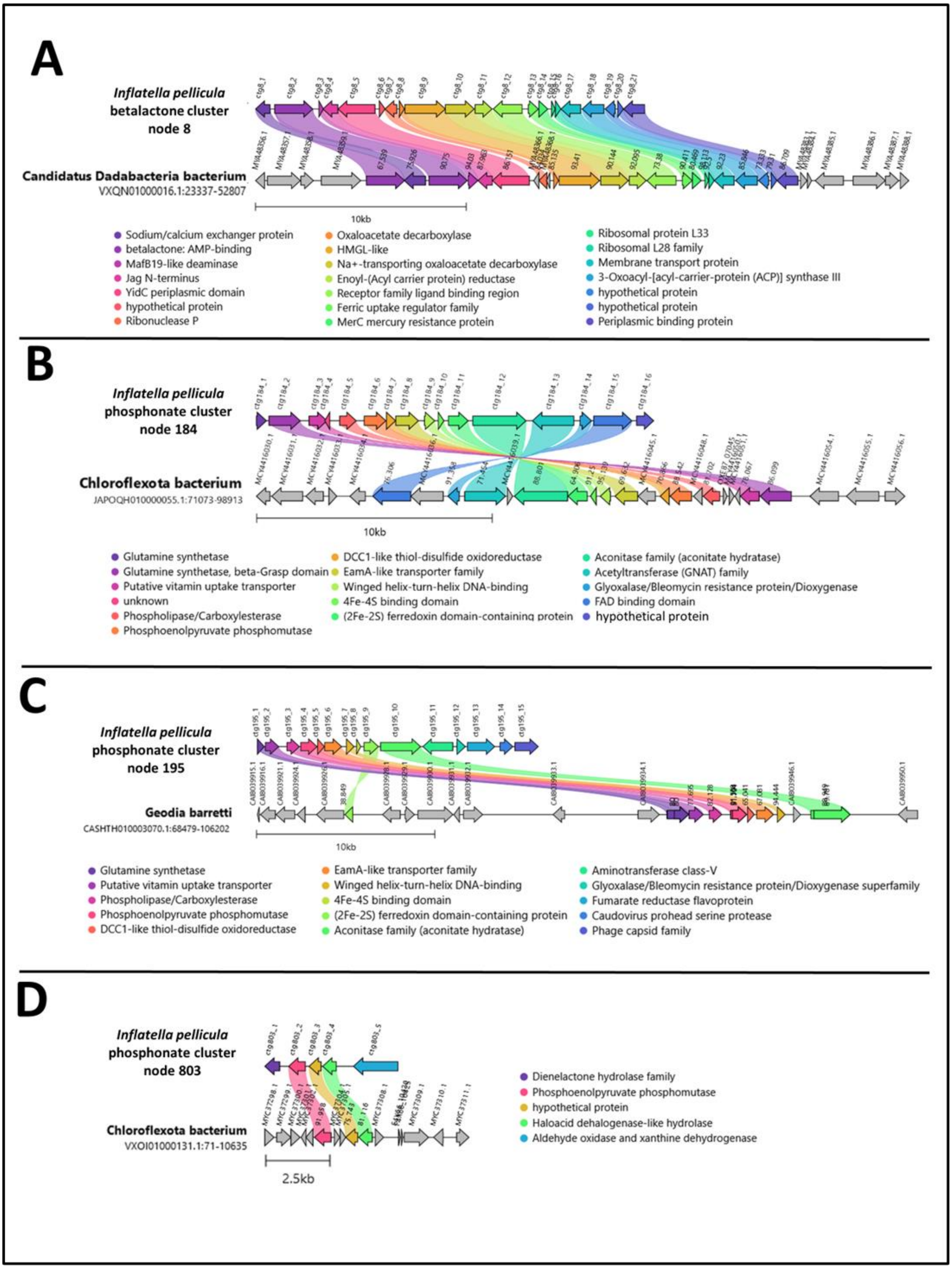

Supplemetary Figure S7: Alignment and annotation of betalactone or phosphonate-encoding secondary metabolism gene clusters from the metagenome of *I. pellicula* and the closest related clusters as identified by cblaster analysis. Where homologous genes were present, percentage identities are indicated, where no homologous genes were present GenBank accession numbers are indicated.

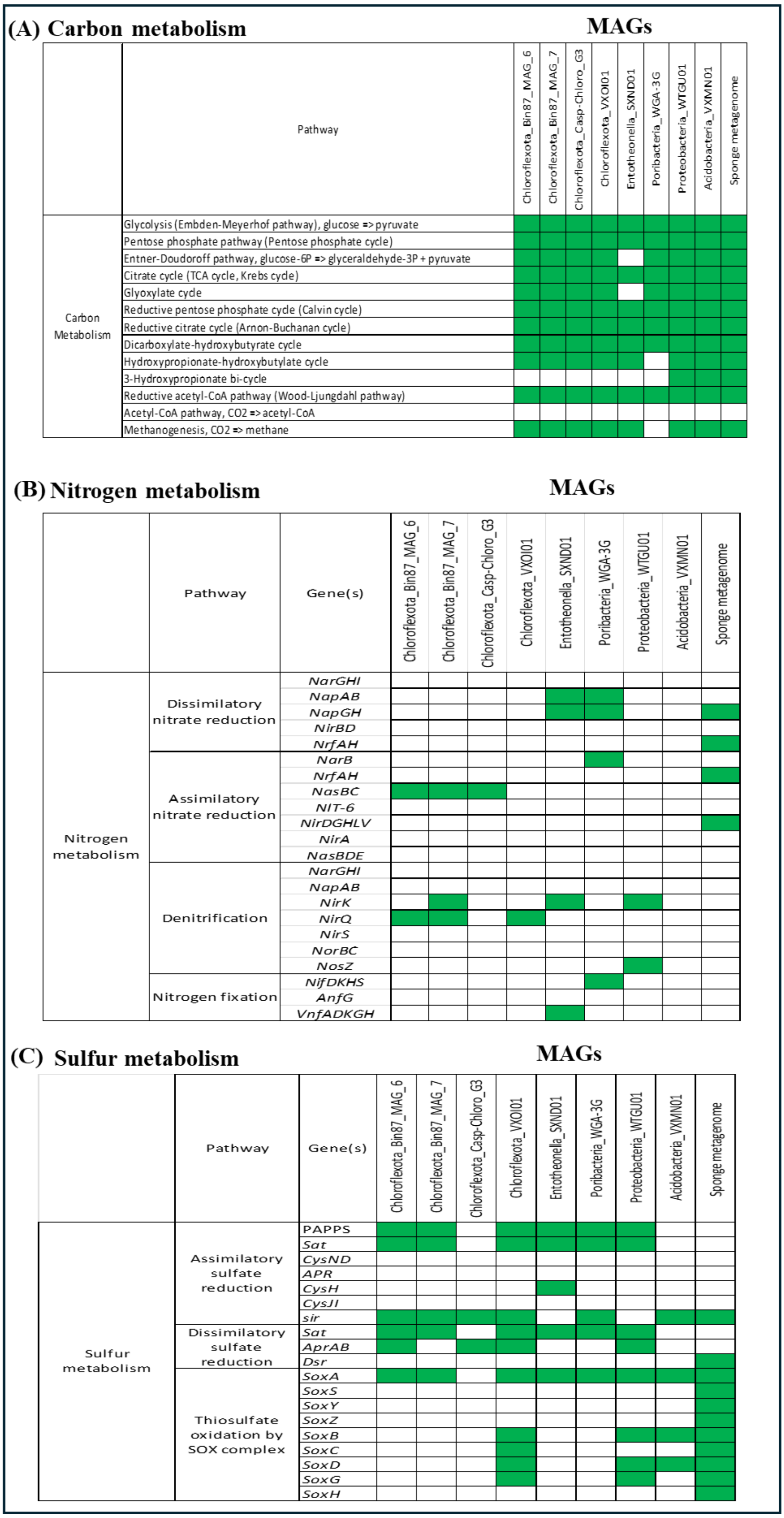
(A) Carbon metabolism
MAGs
Pathway
Chloroflexota_Bin87_MAG_6
Chloroflexota_Bin87_MAG_7
Chloroflexota_Casp-Chloro_G3
Chloroflexota_VXOI01
Entotheonella_SXND01
Poribacteria_WGA-3G
Proteobacteria_WTGU01
Acidobacteria_VXMN01
Sponge metagenome
Carbon Metabolism
Glycolysis (Embden-Meyerhof pathway), glucose => pyruvate
Pentose phosphate pathway (Pentose phosphate cycle)
Entner-Doudoroff pathway, glucose-6P => glyceraldehyde-3P + pyruvate
Citrate cycle (TCA cycle, Krebs cycle)
Glyoxylate cycle
Reductive pentose phosphate cycle (Calvin cycle)
Reductive citrate cycle (Arnon-Buchanan cycle)
Dicarboxylate-hydroxybutyrate cycle
Hydroxypropionate-hydroxybutylate cycle
3-Hydroxypropionate bi-cycle
Reductive acetyl-CoA pathway (Wood-Ljungdahl pathway)
Acetyl-CoA pathway, CO2 => acetyl-CoA
Methanogenesis, CO2 => methane
(B) Nitrogen metabolism
MAGs
Pathway
Gene(s)
Nitrogen metabolism
Dissimilatory nitrate reduction
NarGHI
NapAB
NapGH
NirBD
NrfAH
Assimilatory nitrate reduction
NarB
NrfAH
NasBC
NIT-6
NirDGHLV
NirA
NasBDE
Denitrification
NarGHI
NapAB
NirK
NirQ
NirS
NorBC
NosZ
Nitrogen fixation
NifDKHS
AnfG
VnfADKGH
(C) Sulfur metabolism
MAGs
Pathway
Gene(s)
Sulfur metabolism
Assimilatory sulfate reduction
PAPPS
Sat
CysND
APR
CysH
CysJI
sir
Dissimilatory sulfate reduction
Sat
AprAB
Dsr
Thiosulfate oxidation by SOX complex
SoxA
SoxS
SoxY
SoxZ
SoxB
SoxC
SoxD
SoxG
SoxH

Supplementary Figure S8: (A) Carbon, (B) nitrogen and (C) sulfur metabolism related genes in MAGs assembled from the metagenome of *I. pellicula* and those present in the the wider microbiome. Green-fill boxes indicate presence, empty boxes indicate absence.

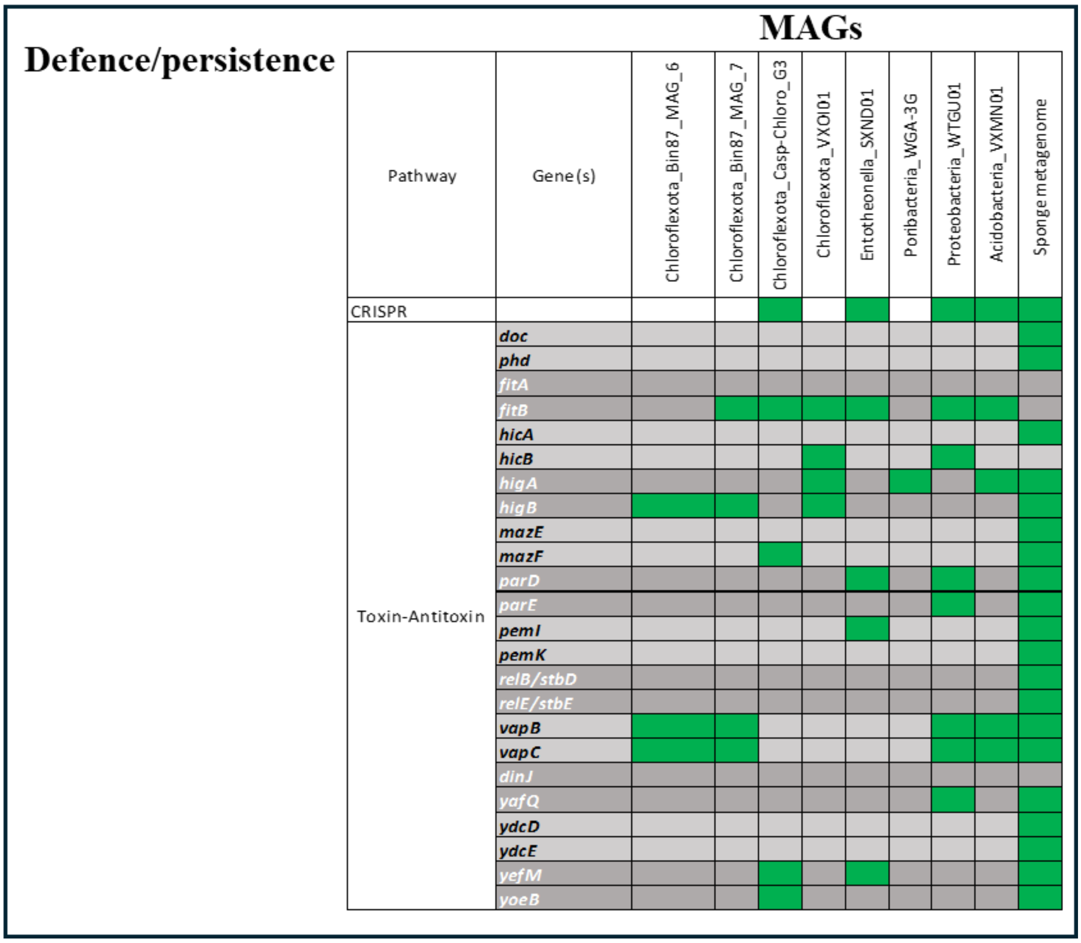

Supplementary Figure S9: Presence or absence of CRISPR genes or toxin/anti-toxin genes in MAGs assembled from the metagenome of *I. pellicula* and those present in the the wider microbiome. Green-fill boxes indicate presence, white or grey-fill boxes indicate absence.

Cluster type	Cluster ID	MAG (this study)	Closest MIBiG Cluster	Compound(s)	Closest cblaster Cluster	Isolation Source of Reference MAG
T3PKS	Node_5	None	BGC0000286 (*Streptomyces* sp. KO-3988)	viguiepinol	VXSQ01000015; MYK49286-MYK49320 (Candidatus *Dadabacteria* bacterium isolate SB0665_bin_2)	*Ircinia ramosa* SAMN12598375
Betalactone	Node_8	None	BGC0000888 (*Bacillus* sp. CS93)	bacilysin	VXQN01000016; MYA48356-MYA48388 (*Dadabacteria* sp.)	*Ircinia ramosa* SAMN12598320
Terpene	Node_43	metaBAT2_bin.008 d__Bacteria;p__Chloroflexota;c__Dehalococcoidia;o__UBA3495;f__UBA3495;g__Bin87;s__	BGC0000648 (*Myxococcus xanthus* DK1050)	carotenoid	VXTR01000068; MXZ91865-MXZ91899 (*Chloroflexota* bacterium isolate SB0666_bin_1)	*Ircinia ramosa* SAMN12598402
RRE-containing	Node_100	None	BGC0002407 & BGC0002621 (*Streptomyces angustmyceticus* NBRC 3934)	angustmycins & aristeromycin	QQVT01000006; RDJ31989-RDJ32011 (*Thermoproteota* archaeon isolate MB_CRA_1)	biofilter metagenome SAMN09708820
RRE-containing	Node_131	CONCOCT_bin.020 d__Bacteria;p__Poribacteria;c__WGA-4E;o__WGA-4E;f__WGA-3G;g__WGA-3G;s__	BGC0002442 (*Bacillus toyonensis* XIN-YC13)	toyoncin	PYIY01000190; RKU09661-RKU09674 (Candidatus *Poribacteria* bacterium isolate PCPOR4)	*Pseudoceratina* sp. SAMN08660835
Terpene	Node_144	metaBAT2_bin.006 d__Bacteria;p__Chloroflexota;c__Dehalococcoidia;o__UBA3495;f__UBA3495;g__Bin87;s__	BGC0000647 (*Rhodobacter sphaeroides* NCIB 8253)	carotenoid	VXTR01000068; MXZ91870-MXZ91896 (*Chloroflexota* bacterium isolate SB0666_bin_1)	*Ircinia ramosa* SAMN12598402
Terpene	Node_157	None	BGC0002283 (*Serratia plymuthica* WS3236)	sodorifen	JAQCFS010000020; MDA0738539-MDA0738567 (*Nitrospirota* bacterium isolate casp40-mb.163)	Caspian sea 50 m depth SAMN32185491
Phosphonate	Node_184	metaBAT2_bin.006 d__Bacteria;p__Chloroflexota;c__Dehalococcoidia;o__UBA3495;f__UBA3495;g__Bin87;s__	BGC0001683 (*Bacillus* sp. 2_A_57_CT2)	N-acyl serinol	JAPOQH010000055; MCY4416030-MCY4416056 (*Chloroflexota* bacterium isolate APA_bin_80 c_000000058053)	*Aplysina aerophoba* SAMN15854948
Phosphonate	Node_195	metaBAT2_bin.008 d__Bacteria;p__Chloroflexota;c__Dehalococcoidia;o__UBA3495;f__UBA3495;g__Bin87;s__	BGC0001683 (*Bacillus* sp. 2_A_57_CT2)	N-acyl serinol	CASHTH010003070; CAI8039915-CAI8039950	*Geodia barretti* SAMEA112361515
Terpene	Node_276	CONCOCT_bin.023 d__Bacteria;p__Chloroflexota;c__Dehalococcoidia;o__UBA3495;f__UBA3495;g__VXOI01;s__	BGC0000648 (*Myxococcus xanthus* DK1050)	carotenoid	DQCW01000160; HCP24202-HCP24225 (*Dehalococcoidia* bacterium isolate UBA9611)	'marine metagenome' SAMN08019616
NRPS-like	Node_312	metaBAT2_bin.011 d__Bacteria;p__Acidobacteriota;c__Acidobacteriae;o__VXMN01;f__VXMN01;g__VXMN01;s__	BGC0001831 (*Myxococcus xanthus* DK 1622)	alkylpyrones	VXMN01000157; MYC81966-MYC81981 (*Acidobacteriota* bacterium isolate SB0661_bin_38)	*Ircinia ramosa* SAMN12598216
Terpene	Node_321	None	BGC0000647 (*Rhodobacter sphaeroides* NCIB 8253)	carotenoid	JACZSN010000017; MCH8868487-MCH8868508 (*Chloroflexota* bacterium isolate B113T1L10)	Mariana trench surficial sediment 10,953m depth SAMN16244448
RiPP-like	Node_340	None	BGC0001485 (*Actinoplanes tsinanensis* CPCC 200056)	chuangxinmycin	JAPOWP010000029; MCY3955416-MCY3955426 (*Nitrospira* sp. isolate COS2_bin_30	*Coscinoderma mathewsi* SAMN15855119
NRPS-like	Node_404	CONCOCT_bin.020 d__Bacteria;p__Poribacteria;c__WGA-4E;o__WGA-4E;f__WGA-3G;g__WGA-3G;s__	BGC0000871 (*Myxococcus xanthus* DK 1622)	VEPE, AEPE, TG-1	VXMD01000032; MYC76008-MYC76026 (Candidatus *Poribacteria* bacterium isolate SB0661_bin_29)	*Ircinia ramosa* SAMN12598206
T3PKS	Node_536	None	BGC0002656 (*Streptacidiphilus oryzae* TH49)	oryzanaphthopyrans, oryzanthrones, chlororyzanthrones	VXVG01000193; MXX21396-MXX21405 (*Cenarchaeum* sp. SB0667_bin_13)	*Ircinia ramosa* SAMN12598443
T3PKS	Node_557	None	BGC0001962 (*Cyanobium* sp. LEGE 06113)	hierridins	JAPOZE010000699; MCY3759575-MCY3759582 (Acidobacteriota bacterium isolate COS4_bin_56)	*Coscinoderma mathewsi* SAMN15855194
Phosphonate	Node_803	CONCOCT_bin.023 d__Bacteria;p__Chloroflexota;c__Dehalococcoidia;o__UBA3495;f__UBA3495;g__VXOI01;s__	BGC0000904 (*Streptomyces rubellomurinus* 5818)	FR-900098	VXOI01000131; MYC372998-MYC37311 (*Chloroflexota* bacterium isolate SB0662_bin_29)	*Ircinia ramosa* SAMN12598263

Supplementary Table S1: smBGCs identified in the metagenome of *Inflatella pellicula* and the closest related clusters, linked to known metabolites in the MIBiG database and clusters where metabolic products are largely unknown, from GenBank.

	Whole Metagenome	B226_SM_ CONCOCT_ bin.001	B226_SM_ CONCOCT_ bin.018	B226_SM_ CONCOCT_ bin.020	B226_SM_ CONCOCT_ bin.023	B226_SM_ CONCOCT_ bin.025	B226_SM_ metaBAT2_ bin.006	B226_SM_ metaBAT2_ bin.008	B226_SM_ metaBAT2_ bin.011
**# contigs**	272426	462	467	706	376	544	295	234	605
# contigs (>= 0 bp)	272426	462	467	706	376	544	295	234	605
# contigs (>= 1000 bp)	63093	462	467	706	376	544	295	234	605
# contigs (>= 10000 bp)	625	33	13	65	51	44	73	78	53
# contigs (>= 100000 bp)	1	0	0	0	0	0	0	1	0
# contigs (>= 1000000 bp)	0	0	0	0	0	0	0	0	0
Largest contig	106607	24740	15425	30719	29654	29910	63971	106607	24654
**Total length**	262348449	2372085	2034616	3869650	2479971	2899894	2515828	2722277	3429276
Total length (>= 0 bp)	262348449	2372085	2034616	3869650	2479971	2899894	2515828	2722277	3429276
Total length (>= 1000 bp)	123554604	2372085	2034616	3869650	2479971	2899894	2515828	2722277	3429276
Total length (>= 10000 bp)	10633876	417622	158344	887521	766069	603150	1386115	1990572	722334
Total length (>= 100000 bp)	106607	0	0	0	0	0	0	106607	0
Total length (>= 1000000 bp)	0	0	0	0	0	0	0	0	0
N50	945	5630	4472	6095	7744	5710	12142	21363	6081
N75	649	3754	3271	3907	4930	3926	6240	9124	4206
L50	70937	139	156	203	106	159	62	39	181
L75	156293	269	288	402	207	314	138	84	348
GC (%)	61.05	62.66	62.13	48.27	62.97	67.58	61.31	59.6	61.6
**Mismatches**									
# N's	129844	380	6650	11090	4060	4520	13210	2440	7030
# N's per 100 kbp	49.49	16.02	31.95	286.59	163.71	155.87	525.08	89.63	205
**Predicted genes**									
# predicted genes (unique)		2466	2052	2899	2476	2820	2409	2604	2980
# predicted genes (>= 0 bp)		2271 + 195 part	1909 + 143 part	2770 + 130 part	2373 + 103 part	2571 + 249 part	2341 + 69 part	2537 + 69 part	2833 + 147 part
# predicted genes (>= 300 bp)		1981 + 180 part	1657 + 126 part	2640 + 127 part	2092 + 86 part	2284 + 225 part	2068 + 61 part	2251 + 64 part	2474 + 142 part
# predicted genes (>= 1500 bp)		224 + 19 part	178 + 10 part	534 + 26 part	218 + 9 part	339 + 35 part	251 + 8 part	265 + 7 part	473 + 30 part
# predicted genes (>= 3000 bp)		13 + 2 part	11 + 1 part	62 + 1 part	12 + 1 part	19 + 1 part	23 + 1 part	22 + 1 part	38 + 3 part

Supplementary Table S2: Sequence assembly statistics of the *I. pellicula* metagenome and of the MAGs assembled from the metagenome.
